# A hybrid RNA-based reporter assay for robust quantification of cytidine deaminase activity

**DOI:** 10.1093/nar/gkag588

**Published:** 2026-06-11

**Authors:** Anna Ligasová, Martina Horejšová, David Friedecký, Eva Pokorná, Pavel Klener, Karel Koberna

**Affiliations:** Institute of Molecular and Translational Medicine, Faculty of Medicine and Dentistry, Palacký University Olomouc, Hněvotínská 5, 779 00 Olomouc, Czech Republic; Institute of Molecular and Translational Medicine, Faculty of Medicine and Dentistry, Palacký University Olomouc, Hněvotínská 5, 779 00 Olomouc, Czech Republic; Laboratory of Inherited Metabolic Disorders, Department of Clinical Chemistry, University Hospital Olomouc, Zdravotníků 248/7, 779 00 Olomouc, Czech Republic; Laboratory of Inherited Metabolic Disorders, Department of Clinical Chemistry, University Hospital Olomouc, Zdravotníků 248/7, 779 00 Olomouc, Czech Republic; Institute of Pathological Physiology, First Faculty of Medicine, Charles University, U Nemocnice 5, 128 53 Prague 2, Czech Republic; Institute of Pathological Physiology, First Faculty of Medicine, Charles University, U Nemocnice 5, 128 53 Prague 2, Czech Republic; Institute of Molecular and Translational Medicine, Faculty of Medicine and Dentistry, Palacký University Olomouc, Hněvotínská 5, 779 00 Olomouc, Czech Republic

## Abstract

We developed a robust, hybrid RNA-based reporter assay for the quantification of cytidine deaminase (CDA) activity, overcoming limitations of current methods often compromised by interferences in crude biological samples. Our two-step method physically separates the enzymatic reaction in cell lysates from a highly specific detection step in a selected and validated CDA-deficient reporter cell line (143B cell line). This design confers exceptional robustness, eliminating the need for sample purification. In the first step, a cell lysate converts 5-fluorocytidine to 5-fluorouridine. After heat inactivation of lysate proteins, 5-fluorouridine is quantified by its incorporation into reporter cell RNA, yielding a highly specific fluorescent signal. The detection limit of a formed product in the assay achieves 2.7 µM. The assay shows good correlation with LC-MS data, and its adaptability to a multi-well plate format makes it an ideal and cost-effective tool for high-throughput screening. We further applied this assay to reveal that CDA-positive cells actively secrete 5-fluorouridine, providing quantitative evidence for a potential therapeutic bystander effect.

## Graphical Abstract

**Graphical Abstract:**
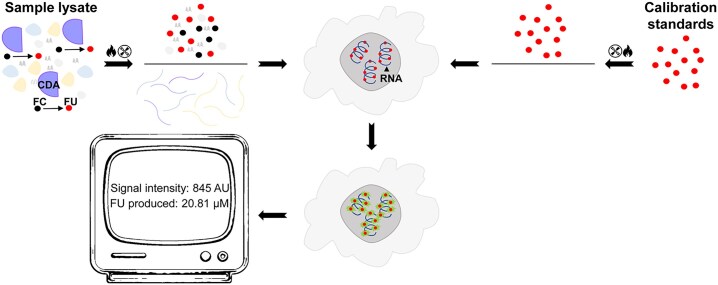

## Introduction

Cytidine deaminase (CDA, EC 3.5.4.5) is a key enzyme in the pyrimidine salvage pathway that catalyses the hydrolytic conversion of cytidine to uridine and deoxycytidine to deoxyuridine [1–3]. A deficiency in CDA can lead to an abnormal accumulation of dCTP, which subsequently slows the progression of DNA replication forks and results in the presence of under-replicated genomic DNA [4]. More recently, Frances and colleagues demonstrated that CDA expression (even independent of its catalytic activity) can influence mitochondrial metabolites, cellular respiration, ATP production, and mitochondrial biogenesis in pancreatic cancer cells [5].

The clinical significance of CDA is considerable. In oncology, CDA plays a crucial role in the metabolism of cytidine analogues, a widely used class of chemotherapeutics for treating various types of cancer, including myelodysplastic syndrome (MDS), acute myeloid leukaemia (AML), pancreatic carcinoma, and lung cancer [6]. High expression and/or activity of CDA can lead to the rapid deamination and inactivation of nucleoside analogues. This reduces both their systemic exposure and therapeutic efficacy in tumour cells. Conversely, reduced CDA activity or its pharmacological inhibition can lead to increased exposure to the active form of the drug, potentially resulting in more severe side effects (e.g. increased toxicity). Typical examples of drugs used in cancer treatment are cytarabine, gemcitabine, and decitabine [7–12].

On the other hand, CDA can also play an activating role. In the case of capecitabine, it is first converted to 5′-deoxy-5-fluorocytidine by the enzyme carboxylesterase. In the next step, CDA deaminates it to 5′-deoxy-5-fluorouridine (dFU) [2, 13, 14]. This compound is then further metabolized in subsequent steps to 5-fluoro-2′-deoxyuridinemonophosphate (FdUMP), which inhibits thymidylate synthase. This inhibition disrupts dTTP synthesis and ultimately leads to the inhibition of DNA replication [15–19]. Furthermore, in the presence of mutagens such as 5-hydroxymethyl-2′-deoxycytidine (5hmdC) and 5-formyl-2′-deoxycytidine (5fdC), their deamination results in the formation of uridine derivatives (5hmdU and 5fdU). These derivatives can then be incorporated into genomic DNA, leading to extensive DNA damage, cell cycle and DNA synthesis arrest, and ultimately, cell death [20, 21].

Current methods for measuring CDA activity each have significant limitations. High-end techniques such as liquid chromatography coupled with mass spectrometry (LC-MS) provide high accuracy but are also expensive, technically demanding, and unsuitable for high-throughput screening [22, 23]. More recently, highly sensitive real-time assays using custom-synthesized fluorescent probes have been described [24, 25]; however, their reliance on complex, non-commercial substrates can limit their widespread adoption. Simpler and more cost-effective techniques like traditional spectrophotometry are available, but their direct application is often limited by interferences from other components in crude biological samples that require purification [19]. Radiometric assays, although sensitive, are limited by safety concerns and the production of radioactive waste [26–29].

Methods based on monitoring CDA activity directly in cells have also been developed [2, 30]. One example employs substrates like 5-fluorocytidine (FC), which is converted by CDA to 5-fluorouridine (FU) and subsequently detected after its incorporation into RNA [2]. Although these cell-based approaches offer a functional perspective, their main disadvantage is the difficulty of making quantitative comparisons between different types of cells or tissues. The resulting signal is influenced not only by CDA activity but also by a whole range of other cellular processes that differ between samples—for instance, the efficiency of nucleoside transporters or the activity of subsequent metabolic pathways. Moreover, as studies show, the toxicity of the FU product is complex in itself, involving disruption of pre-mRNA splicing [31] and RNA modifications like pseudouridylation [32], which further complicates the interpretation of results when comparing different cellular systems.

Thus, a critical need remains for a method that combines affordability with the robustness to function reliably in complex biological matrices. In this study, we address this challenge by developing and validating a novel, hybrid RNA-based reporter assay for the robust quantification of CDA activity. Our method decouples the enzymatic reaction, which occurs in a cell lysate, from a highly specific detection step utilizing a carefully selected and validated CDA-deficient reporter cell line. This approach provides a powerful and robust tool for the quantitative comparison of CDA activity across diverse biological samples, eliminating key sources of variability inherent in other methods.

## Materials and methods

### Cell lines and culture conditions

The human cell lines, including osteosarcoma 143B (ATCC, CRL-8303), lung carcinoma A549 (DSMZ, ACC 107), cervical adenocarcinoma HeLa (ATCC, CCL-2), hepatocellular carcinoma HepG2 (ATCC, HB-8065), normal foetal lung fibroblasts WI-38 (ATCC, CCL-75), normal foetal lung fibroblasts IMR-90 (ATCC, CCL-186), and hTERT immortalized retinal pigment-epithelial cells (hTERT RPE-1, a gift from Prof. David Staněk, Institute of Molecular Genetics CAS, Prague) were used in this study. The 143B cell line was selected as the reporter line for the assay. The HeLa, A549, 143B, and hTERT RPE-1 cells were cultivated in Dulbecco’s modified Eagle’s medium (DMEM, ThermoFisher Scientific, 52100-039) supplemented with 10% foetal bovine serum (Capricorn, CP21-4327), 3.7 g/l of sodium bicarbonate (Merck, S3817), and 50 µg/ml of gentamicin (Merck, G1272). HepG2, WI-38, and IMR-90 cells were cultivated in Eagle’s minimum essential medium (EMEM, Merck, M1018) supplemented with 20% foetal bovine serum, 3.7 g/l of sodium bicarbonate, and 50 µg/ml of gentamicin. The cells were cultivated at 37°C in a humidified atmosphere containing 5% CO_2_. All cell lines were regularly tested for mycoplasma contamination by PCR and enzymatic detection [33].

### PDX model

The patient-derived xenograft (PDX) model VFN-AML2 used in this study had been derived from a patient with AML as part of other research projects approved by Ethics Committee of the General University Hospital Prague under approval number 48/18 Grant AZV VES 2019 VFN at the Institute of Pathology, First Faculty of Medicine, Charles University, Prague, as described previously (for example [34]). The animal study protocol was first approved by the Institutional Review Board of the First Faculty of Medicine, Charles University, and subsequently by the Ministry of Education, Youth, and Sports of the Czech Republic and approved on 22 April 2022 (until December 2025) under the number MSMT-8820/2022–3. The study was approved by the institutional Ethics Committee (48/18). NOD·Cg-Prkdcscid Il2rgtm1Wjl/SzJ mice (further NSG mice) purchased from the Jackson Laboratory (Bar Harbor, ME, USA) were used for all *in vivo* experiments. Animals were housed and maintained in a pathogen-free environment in individually ventilated cages and provided with sterilized food and water. The PDX cells were subcutaneously injected into adult female NSG mice. Once the tumours reached a defined size (i.e. 2 cm), mice were sacrificed, tumours were excised, and cytoplasmic lysates were prepared for further analysis.

### Preparation of cytoplasmic lysates

Cells were cultured to a confluency of 50%–70% in 10 cm diameter dishes. The cell monolayer was washed three times with ice-cold 1× PBS, followed by a single rapid wash with 5 ml of ice-cold buffer A (10 mM Tris–HCl, pH 7.5; 10 mM KCl). For lysis, 2 ml of ice-cold hypotonic buffer B (10 mM Tris–HCl, pH 7.5; 10 mM KCl; 0.5 mM ethylenediaminetetraacetic acid, supplemented with a protease inhibitor cocktail, Merck, P2714) was added to the dish. The dishes were incubated on ice for 20 min with occasional swirling to allow for cell swelling. The swollen cells were then scraped using a cell scraper and transferred to a pre-chilled Dounce homogenizer (Merck, D8938) and homogenized (30 times). The homogenate was transferred into a 2-ml tube and centrifuged for 10 min at 12 000 × *g* and 4°C. The liquid cytoplasmic fraction was transferred into a new tube and used for analysis of CDA activity or aliquoted and stored at −80°C until used.

In the case of tumours prepared from PDX model, cytoplasmic lysates were prepared as follows: the excised tumour was washed in 1× PBS buffer and disintegrated through a 70 μm cell strainer. Around 20 × 10^6^ cells were used per sample. Cell suspension was centrifuged (100 × *g*, 5 min, 25°C) and the supernatant was removed. The ice-cold 1× PBS was added, cell pellet was re-suspended, and the samples were centrifuged again. This washing step was repeated once again. After buffer removal, the centrifuge tube with the cell pellet was put on ice, and 2 ml of the ice-cold buffer B was added. Cell pellet was re-suspended. The remaining steps were the same as in the case of cell lysates from cell cultures described earlier.

The total protein concentration of the lysates was determined using BCA protein assay (Thermo Fisher Scientific, 23225) to allow for normalization of all samples to a standard concentration for the enzymatic assay. The protocol recommended by the assay manufacturer was used. For HeLa cells, this lysis protocol typically yielded ~132.3 pg of cytoplasmic protein per cell.

### Hybrid CDA activity assay using cell lysates

#### The determination of CDA activity using a hybrid two-step method

##### Step 1: Enzymatic reaction

The cytoplasmic lysate was first diluted with buffer B to a standard protein concentration of 200 µg/ml. The cell lysate was incubated with FC (Biosynth, NF04135) as a substrate at a final concentration of 1–2 mM. The reaction was carried out at 37°C for 1 h. To stop the enzymatic reaction, the mixture was subsequently heated to 90–96°C for 10–20 min to denature all enzymes. The denatured proteins were pelleted by centrifugation (12 000 × *g*, 10 min, 4°C), and the supernatant containing the reaction product, FU, was transferred to a new tube. At this stage, samples could be frozen for later analysis.

##### Step 2: Reporter-based detection

The 143B reporter cells were seeded onto round, 12-mm, glass coverslips in multi-well plates and allowed to attach. The heat-inactivated supernatant from Step 1 was mixed 1:1 with 2× concentrated culture medium (DMEM). This mixture was then added to the reporter cells and incubated for 1 h under standard culture conditions (37°C, 5% CO₂). The 1:1 mixing strategy was employed to maintain near-physiological osmotic conditions for the reporter cells while allowing a large volume of the experimental sample to be tested, thereby maximizing the assay’s sensitivity.

#### Assay for CDA product secreted into culture medium

To measure the CDA product secreted by live cells, various cell lines were seeded in multi-well plates. The following day, the culture medium was replaced with the fresh medium containing 100 µM FC and incubated for 1 h. After the incubation, the conditioned medium was harvested and centrifuged to remove any detached cells. The supernatant was then mixed 1:1 with 1× concentrated culture medium (DMEM) and added to the pre-seeded 143B reporter cells for 1 h. The subsequent detection of incorporated FU was performed using the immunofluorescence protocol described below. To ensure comparability, the results of these experiments were normalized to 1 × 10^6^ cells. The cell count was determined using a combined method; cell nuclei were first identified in images using ilastik software [35] employing machine learning, and the data were then refined by manual annotation of ~100 nuclei to distinguish individual cells from clumps, enabling the final calculation of the total cell number based on signal intensity and area.

### FU calibration standards

To quantify the amount of FU produced, corresponding sets of calibration standards were prepared. For the hybrid lysate assay, a set of standards was prepared using serial dilutions of FU (Biosynth, NF04076), from 160 µM down to 5 µM, in the same hypotonic buffer used for the lysates (buffer B). These standards were processed through the same heat inactivation and incubation steps with the reporter cells as the experimental samples. In the case of limit of detection (LOD) calculation, standards with serially diluted FU (concentration range was from 50 to 1.5625 µM) were used. These standards were then added to the reporter cells. For the assay of FU secreted into the medium, a separate calibration curve was prepared by diluting FU to final concentrations of 0 to 100 µM in fresh cell culture medium. These standards were then added to the reporter cells alongside the conditioned medium samples.

### Fluorescent ammonia assay

For comparative purposes, ammonia production was also measured using the commercial ammonia assay kit based on the *o*-phthalaldehyde method (MAK310, Merck) according to manufacturer’s protocol. To avoid interference from primary amines, tested cells (HeLa and A549) were lysed in 0.1× PBS instead of the Tris-based buffer B. Due to the high background fluorescence generated by the crude lysates, the total protein concentration had to be reduced to 50 µg/ml to remain within the assay’s linear range. Lysates were incubated with 2 mM FC or with deionized water instead of FC (control samples) for 15 min in the dark, and fluorescence was measured (excitation at 360 nm, emission at 450 nm). Simultaneously, calibration standards from supplied ammonium chloride were prepared following the manufacturer’s protocol. From the measured data, the calibration curve was constructed using linear regression function with the interpolated X values using GraphPad Prism 6 software.

### LC-MS

CDA activity in cytoplasmic lysates of PDX model was measured by LC-MS following the methodology detailed in our previous work [30].

### SDS–PAGE, western blot

The SDS–PAGE and western blots were performed according to [36, 37]. In the experiments, 5, 10, or 20 µg of the total protein was used. Precision Plus Protein Dual Colour Standards were used as a protein marker (Bio-Rad, 1610374). The staining with Ponceau S (Merck, P3504) was used as a loading control [38–40]. Membranes were incubated with a primary antibody against CDA (clone D-5, Santa Cruz Biotechnology, sc-365292), followed by a peroxidase-labelled secondary antibody (Jackson ImmunoResearch Europe, 115-035-146). The data were evaluated using SIMCIM 1.01 tools software [2, 41] and Microsoft Excel software [2, 36, 37]. At first, signal from Ponceau S labelling was calculated for every whole lane. Then, the signal of the band corresponding to the CDA was measured. Finally, the CDA signal was normalized to the total protein content.

### Apoptosis detection

To evaluate the apoptotic and necrotic cells, Annexin V-FITC apoptosis detection kit (Merck, APOAF) was used according to the manufacturer’s instructions. Either control cells or cells treated with 100 μM FC for 1 h were incubated in the Petri dish with the glass bottom (Mattek, P35G-1.5-14-C). After brief wash with 1× binding buffer provided in the kit, 500 μl of the solution of Annexin V (5 μl of ∼50 μg/ml stock solution) and propidium iodide (10 μl of 100 μg/ml stock solution) was added to every tested Petri dish with cells. For the cell nuclei labelling, Hoechst 33342 (Thermo Fisher Scientific, H1399) was added to the prepared solution as well (2 μl of 10 mM stock solution). Samples were incubated for 10 min in the dark and immediately analysed by fluorescence microscopy.

### Calculations

The concentration of FU derived from the calibration curve was used to determine the original concentration in the lysate supernatant and the total amount of FU produced during the enzymatic reaction.

#### Calculation of the original FU concentration

The concentration of FU in the original, undiluted lysate supernatant (*C*_*original*_) was calculated by correcting the concentration measured from the calibration curve (*cal_conc*) for all subsequent dilution factors (1):


(1) \begin{eqnarray*}
{{\mathrm{\mathit{ C}}}_{{\mathrm{\mathit{ original}}}}} = {\mathrm{\mathit{ cal}}}\_{\mathrm{\mathit{ conc}}} \times {\mathrm{\mathit{ dil}}}\_{\mathrm{\mathit{ medium}}} \times {\mathrm{\mathit{ dil}}}\_{{\textit{buffer}}}.
\end{eqnarray*}

Where *cal_conc* is in µM, *dil_medium* is the 1:1 dilution of sample lysate with the culture medium (factor of 2), and *dil_buffer* is the initial dilution of the lysate (e.g. a factor of 1 or 10).

#### Calculation of the total amount of FU produced

The total amount of FU produced (*nFU*) was then calculated from its concentration (*C*_*original*_) and the initial reaction volume (*V*_*lysate*_) (2):


(2) \begin{eqnarray*}
{\mathrm{\mathit{ nFU}}} = {{\mathrm{\mathit{ C}}}_{{\mathrm{\mathit{ original}}}}} \times {{\mathrm{\mathit{ V}}}_{{\mathrm{\mathit{ lysate}}}}}.
\end{eqnarray*}

Where variables are in consistent units (e.g. amount in nmol, concentration in nM, and volume in l). This calculated amount was used for subsequent comparisons, such as the analysis of the secreted FU fraction.

### Immunofluorescence

Following incubation with samples or standards, the reporter cells on coverslips were washed three times with 1× PBS. The cells were then fixed with 2% formaldehyde (Merck, 818715) for 10 min. For permeabilization, cells were treated with 0.2% Triton X-100 (Merck, X100) for 10 min and washed again with 1× PBS. Blocking was performed by incubating the coverslips on droplets of TNTB buffer [25 mM Tris–HCl, pH 7.5; 150 mM NaCl; 1% BSA (Merck, A7906); 50 mM glycine (Carl Roth, 0079.4); 0.1% Tween 20 (Merck, P9416) for 10 min at room temperature (3×)]. The coverslips were then incubated on a 20 µl droplet of TNTB containing a primary anti-BrdU antibody (clone BU-33, cat. no. B2531, Merck, 1:300 dilution, which cross-reacts with FU) for 30 min. After incubation, the coverslips were washed four times with TNTB buffer, with the final wash lasting 10 min. Subsequently, the coverslips were incubated on a 20 µl droplet of TNTB containing an Alexa Fluor 488-conjugated goat anti-mouse secondary antibody (1:100 dilution, Jackson ImmunoResearch Europe, 115-545-146) and DAPI (10 µM, Thermo Fisher Scientific, D3571) for 30 min in the dark. This was followed by three 5-min washes in TNTB and three washes in TN buffer (25 mM Tris–HCl, pH 7.5; 150 mM NaCl). Finally, the coverslips were rinsed for 10 s in TN buffer and mounted onto a microscope slide using a glycerol-based mounting medium containing DABCO (Merck, D27802) as an anti-fade agent.

### Image acquisition and analysis

#### Microscopy and image acquisition

Fluorescence images for both the CDA activity assay and apoptosis detection were acquired using an Olympus IX83 microscope equipped with a UPLFLN 2PH 10×/0.30 objective (CDA activity assay) or with a LUCPLFLN PH 20×/0.45 objective (apoptosis detection) by a Zyla camera (Andor) with a resolution of 1024 × 1024 pixels. Data were captured using Olympus cellSense Dimension 2.3 software [33, 41]. For each experimental sample, multiple random images were captured to ensure representative sampling.

#### Image analysis

##### Analysis of FU incorporation (CDA activity assay)

For the quantification of incorporated FU, an area-based analysis with precise local background subtraction was employed. This approach used the 143B cell line as a reference, allowing the segmentation model to be trained once and applied consistently across all samples, ensuring robust evaluation without the need for cell-type-specific retraining. For each sample, six random images were evaluated across four independent replicates. At first, primary binary masks defining the total nuclear area were generated in ilastik 1.4.0 software [35] using the *Pixel Classification* module. The model was trained to reliably distinguish DAPI-stained nuclei from the background. Then, the masks were imported into CellProfiler 4.2.5 [42, 43]. To prevent signal interference from optical scattering (fluorescence halo), the nuclear masks were dilated by 3 pixels, and the inverse of these expanded masks was used to define the pure background area. The mean fluorescence intensity of the FU signal was then measured separately within the original nuclear masks and the pure background masks. The final net signal for each image was calculated in Microsoft Excel 2013 by subtracting the mean background intensity from the mean nuclear intensity. This value was subsequently converted to FU concentration using linear regression analysis of the calibration standards, as described in the “Statistical analysis” section. The mean fluorescence intensity of the FU signal was measured in arbitrary units (AU).

##### Apoptosis and cell death evaluation

At least 4500 cells per condition were evaluated across three independent experiments. Image analysis was performed using ImageJ 1.54f software [44]. Cell nuclei labelled with Hoechst 33342 were identified to determine the total cell count. The presence of Annexin V and PI signals was subsequently quantified to calculate the fraction of apoptotic (Annexin V-positive), necrotic (PI-positive), or late apoptotic (double-positive) cells.

### Statistical analysis

Statistical analyses and tests were performed using SigmaPlot 11.0 and GraphPad Prism 6 software; the plots were generated using GraphPad Prism 6 software. Comparisons of individual cell line or PDX model activities against a theoretical mean of zero were conducted using a one-sided, one-sample t-test or a Wilcoxon signed-rank test, based on data distribution. Comparisons between two experimental groups were conducted using either a paired t-test for related samples or an independent t-test for unrelated samples. Linear regression analysis was used to evaluate the dose-response calibration curves. The relationship between the results obtained from the hybrid assay and the LC-MS method was assessed using the Pearson correlation coefficient. Adobe Photoshop was used to prepare the final figures. Unless otherwise stated, all experiments were conducted in three independent replicates, and graphical data are presented as mean ± standard deviation (SD) to illustrate the variability of the individual experimental replicates. For the presentation of dose-response relationships and calibration curves (such as the LOD calibration and the cell line sensitivity comparison), data are expressed as the mean ± standard error of the mean (SEM). The rationale for using SEM in these specific instances was to reflect the precision of the estimated mean response at each concentration. A *P*-value of <.05 was considered statistically significant.

## Results

### A novel hybrid assay for the sensitive detection of CDA activity

Our primary objective was to develop a rapid, cost-effective, and easily reproducible assay for CDA activity measurement that enables robust quantitative comparisons among samples. Currently, methods based on cellular models are particularly attractive due to their inherent high specificity, which makes them robust against interferences common in crude biological samples. However, these direct assays are often limited to qualitative or semi-quantitative assessments, as their results can be skewed by cell-type-specific differences in substrate transport and subsequent product metabolism, making direct comparisons between different cell lines or tissues unreliable.

**Figure 1.**
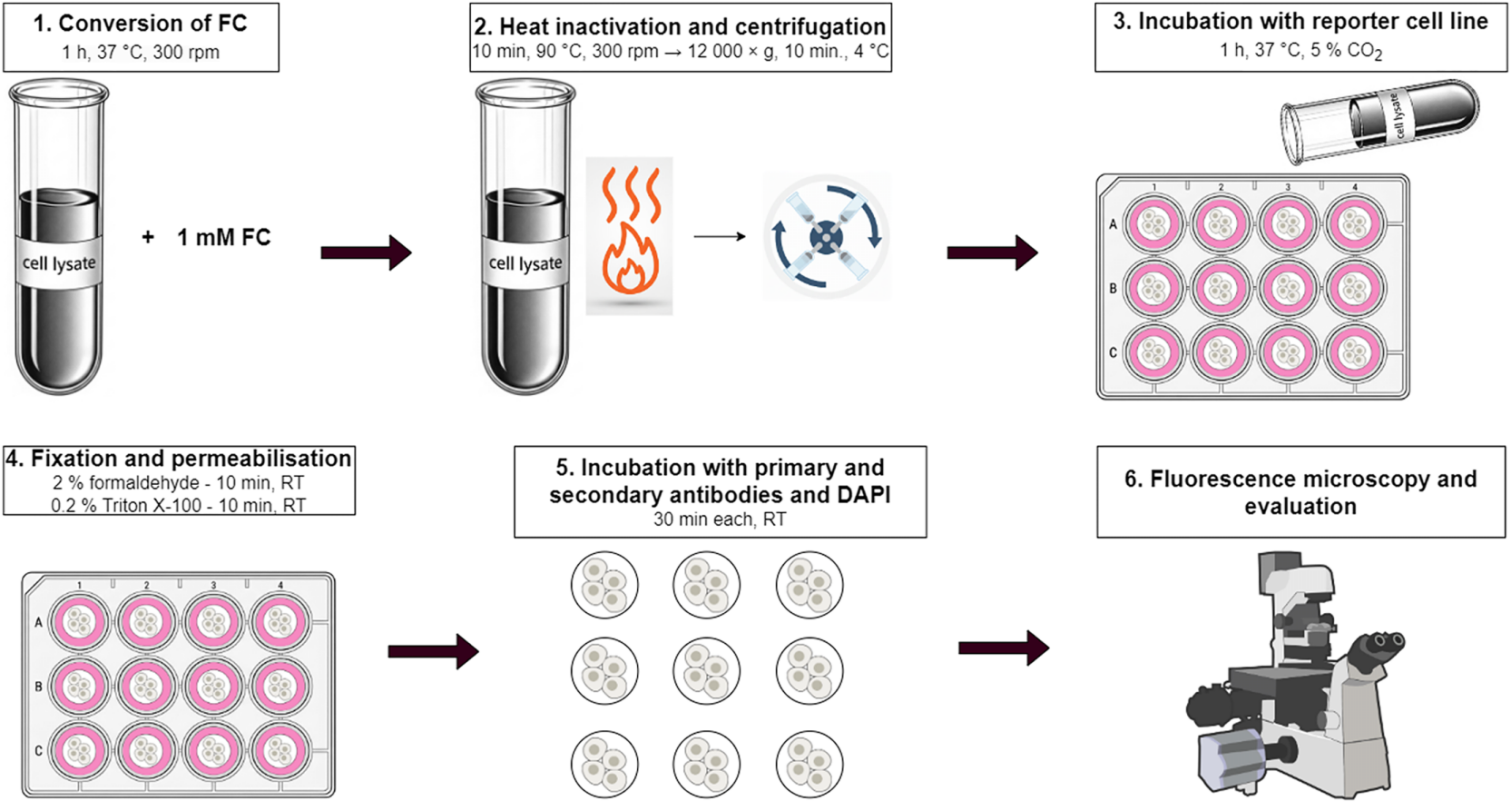
Schematic overview of the hybrid two-step assay for CDA activity measurement. The diagram illustrates the step-by-step workflow of the method. Top panels (steps 1–2): The enzymatic conversion of FC to FU by CDA within the crude cell lysate, followed by heat inactivation and centrifugation to precipitate proteins and eliminate matrix interferences. Top panel (step 3) and bottom panels (steps 4–6): The detection phase, where the cleared, FU-containing supernatant is applied to the CDA-deficient 143B reporter cells. The cells incorporate FU into newly synthesized RNA, which is subsequently visualized using immunofluorescence, quantified by microscopy, and converted to FU concentration using a pre-established calibration curve. White circles (steps 3, 4, and 5) represent 12 mm glass cover slips with cells on their surface, placed inside the plate (steps 3 and 4) or handled individually (step 5). However, it is possible to handle glass coverslips, e.g. in Petri dishes instead of plates.

To overcome these limitations while retaining the specificity of a cell-based readout, we developed a novel two-step hybrid assay. The core principle of this method is the physical separation of the enzymatic reaction from the cellular detection step. By performing the enzymatic conversion *in vitro* within the cell lysate, we effectively decouple the measurement of CDA activity from the confounding influence of cellular transport mechanisms. This ensures that the generated FU signal results strictly from the *de novo* conversion of the added substrate by the extracted enzyme. As illustrated in Fig. 1, the workflow begins with the enzymatic conversion of FC to FU by CDA in a cell lysate. The reaction is then terminated by heat inactivation. After centrifugation, the amount of generated FU is quantified using a CDA-deficient reporter cell line (143B cell line), which incorporates the FU into its RNA for subsequent immunofluorescent detection. To demonstrate the validity of this approach, we first sought to select and validate the optimal reporter cell line for the assay.

### Selection and validation of the optimal reporter cell line

**Figure 2.**
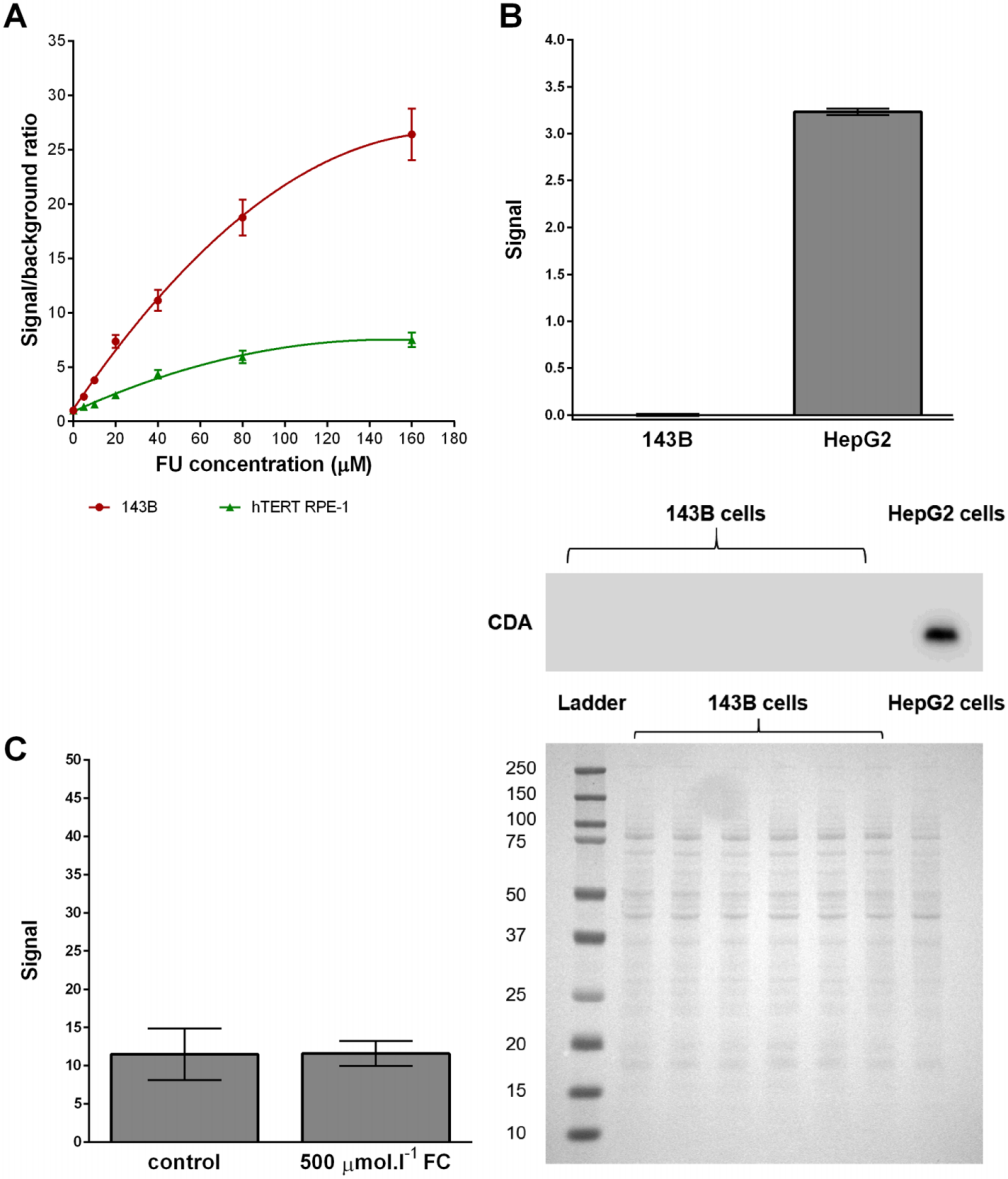
Selection and validation of the 143B reporter cell line. (**A**) Comparison of the dose-dependent signal response between 143B and hTERT RPE-1 reporter cell lines after incubation with known concentrations of FU. The data are shown as the mean ± SEM, *n* = 6. The steeper curve for the 143B cells demonstrates their superior sensitivity and a higher signal-to-noise ratio. (**B**) Western blot analysis of CDA protein expression in 143B cells. No detectable CDA protein was found in cytoplasmic lysates from 143B cells (*n* = 3), in contrast to the strong signal in the positive control, HepG2 cells (*n* = 3). The data are shown as the mean ± SD. A representative western blot and the corresponding Ponceau S staining, used as a loading control, are shown below the graph (all individual biological replicates and uncropped blots are available in the Supplementary Data—Originals of Western blots file). (**C**) Functional test for endogenous CDA activity. 143B cells were incubated in medium with or without (control) 500 µM FC and fluorescence detection of FU was performed. The absence of a significant fluorescence signal in the presence of FC confirms the lack of functional CDA. The data are shown as the mean ± SD, *n* = 6.

A critical component of our hybrid assay is a reporter cell line that lacks endogenous CDA activity, is adherent, and provides a robust, quantifiable signal. We began by evaluating several potential candidates. Primary cell lines, such as IMR-90 and WI-38, while previously reported to have undetectable CDA protein and activity [30], were deemed unsuitable for a standardized, long-term assay due to their limited lifespan caused by replicative senescence. We next considered the hTERT-immortalized RPE-1 cell line. Although this cell line is immortal and lacks detectable CDA activity [30], our analysis revealed that it exhibits a relatively low signal-to-noise ratio. As demonstrated in Fig. 2A, our analysis revealed that the hTERT RPE-1 cell line exhibits a relatively low signal-to-noise ratio, especially at lower substrate concentrations, which compromises reliable quantification in this range. This low sensitivity, combined with the complexities of handling a genetically modified cell line, led us to seek a more suitable candidate. In contrast, the 143B osteosarcoma cell line emerged as an excellent alternative (Fig. 2A).

To confirm suitability of 143B cells, we performed a two-step validation. First, at the molecular level, we demonstrated the absence of any detectable endogenous CDA protein using western blot, in stark contrast to the high-expressing HepG2 positive control (Fig. 2B). Second, we confirmed this finding with a functional assay [2]. Incubation of 143B cells with 500 µM FC did not result in any significant fluorescence signal above background (Fig. 2C, *P* = .9492, independent t-test). These results definitively established the 143B line as a suitable reporter system lacking detectable CDA protein and activity. Crucially, a direct comparison of performance confirmed the superiority of the 143B line. As shown visually in Fig. 2A, the 143B line provided a signal-to-background ratio that was ~67% higher than that of the hTERT-RPE-1 cell line (*P* = .039, paired t-test). To establish its utility for quantification, we analysed its dose-dependent response to FU. A linear regression analysis of the 0–80 µM range confirmed a very strong and statistically significant linear relationship (*R*² = 0.96, *P* < .001), confirming that the 143B reporter system can be used for accurate quantification of FU.

**Figure 3.**
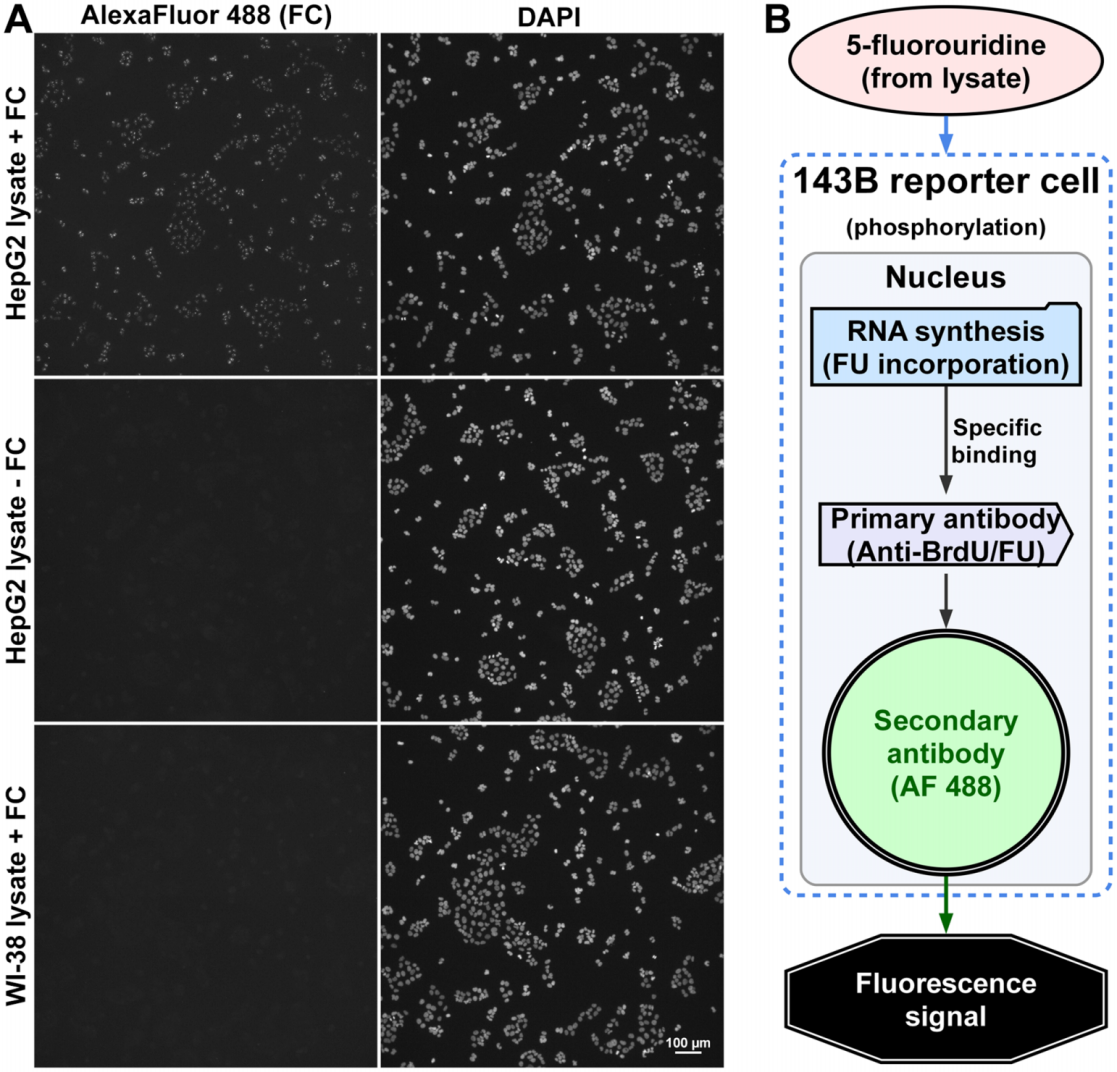
Visual validation of the 143B cellular detection step and schematic depiction of detection steps. (**A**) Representative immunofluorescence images of 143B reporter cells verifying the assay’s specificity. The 143B cells were treated with a CDA-positive cell lysate (HepG2 cells) incubated with the 2 mM FC substrate, showing significant nuclear FU incorporation, or with a CDA-positive cell lysate (HepG2 cells) without the FC substrate, confirming that the crude lysate matrix itself does not generate unspecific background fluorescence, or with a CDA-negative cell lysate (WI-38 fibroblasts) incubated with 2 mM FC, demonstrating the absence of signal and confirming enzyme specificity. DAPI was used to label whole nuclei. Incorporated FU was detected by indirect immunofluorescence (primary anti-BrdU antibody reacting also with FU followed by secondary antibody with AlexaFluor 488 fluorochrome). (**B**) A schematic cartoon illustrating the principle of the reporter assay inside the 143B cells. FU generated in the lysate is taken up by the 143B cells and incorporated into newly synthesized RNA. This incorporated FU is subsequently detected using a primary anti-BrdU/FU antibody and an Alexa Fluor 488-conjugated secondary antibody, yielding a quantifiable fluorescent signal localized in the nucleus.

The assay’s specificity and background levels were visually validated by immunofluorescence images of the 143B reporter cells (Fig. 3A). Images were acquired from an experiment with 143B cells treated with a CDA-positive cell lysate containing the FC substrate, with a CDA-negative cell lysate containing FC, and with a CDA-positive lysate lacking the FC substrate. A mechanistic cartoon of the detection steps is provided in Fig. 3B.

Next, we evaluated the assay’s sensitivity at low FU concentrations. First, we functionally tested the reporter system’s response to a concentration of 3.125 µM FU. The resulting net signal, after background subtraction, was confirmed to be statistically significantly different from zero (one-tailed one-sample t-test, *P* = .0096), demonstrating that this concentration is reliably detectable. To precisely quantify this sensitivity, we then formally calculated the LOD of the formed product (FU) based on the standard deviation of blank samples and the slope of the calibration curve. We assumed that a measurable signal is above the background if the signal is equal to or greater than the mean of the background plus 3 standard deviations (3).


(3) \begin{eqnarray*}
\mathit{mean~signal} &=& \mathit{mean~signal~of~bkg.}\ + \left( 3 \times \mathit{SD}_{\mathit{bkg.}} \right).
\end{eqnarray*}

The calculated LOD signal was equal to 30.71 AU. The LOD concentration was calculated from the calibration curve (Supplementary Fig. S1) using a linear regression function with the interpolated X values using GraphPad Prism 6 software. This analysis yielded a calculated LOD of 2.7 µM. The successful detection of 3.125 µM FU in our functional test is therefore in excellent agreement with this calculated limit, providing robust validation for the assay’s sensitivity in the low micromolar range.

### The hybrid assay accurately quantifies a wide dynamic range of CDA activity

With the 143B reporter system validated, we next optimized the conditions of the enzymatic reaction to accurately measure CDA activity across a wide spectrum of biological samples. A key objective was to reliably detect even extremely low activity, as seen in cell lines like A549 cells. We determined that a high total protein concentration in the lysate, specifically 200 µg/ml, was necessary to generate a robust signal from such low-activity samples. To prevent substrate depletion by highly active lysates under these conditions, a saturating concentration of 2 mM FC was used for the 1-h reaction. The dual-analysis strategy was implemented to ensure the robust measurement of a wide spectrum of CDA activities, particularly for samples with very low enzymatic output. By analysing each sample both undiluted (200 µg/ml protein) and at a 10-fold dilution (effectively 20 µg/ml protein), we ensured that at least one measurement would fall within the validated linear range of the reporter system, which extends up to 80 µM (Supplementary Fig. S2). As demonstrated (Supplementary Fig. S2), high-activity samples like HeLa or HepG2 often produce signals exceeding the upper limit of the calibration range at the standard protein concentration, requiring dilution to yield accurate quantitative data. Conversely, for low-activity cell lines such as A549, the higher protein input is necessary to generate a signal clearly distinguishable from the baseline.

**Figure 4.**
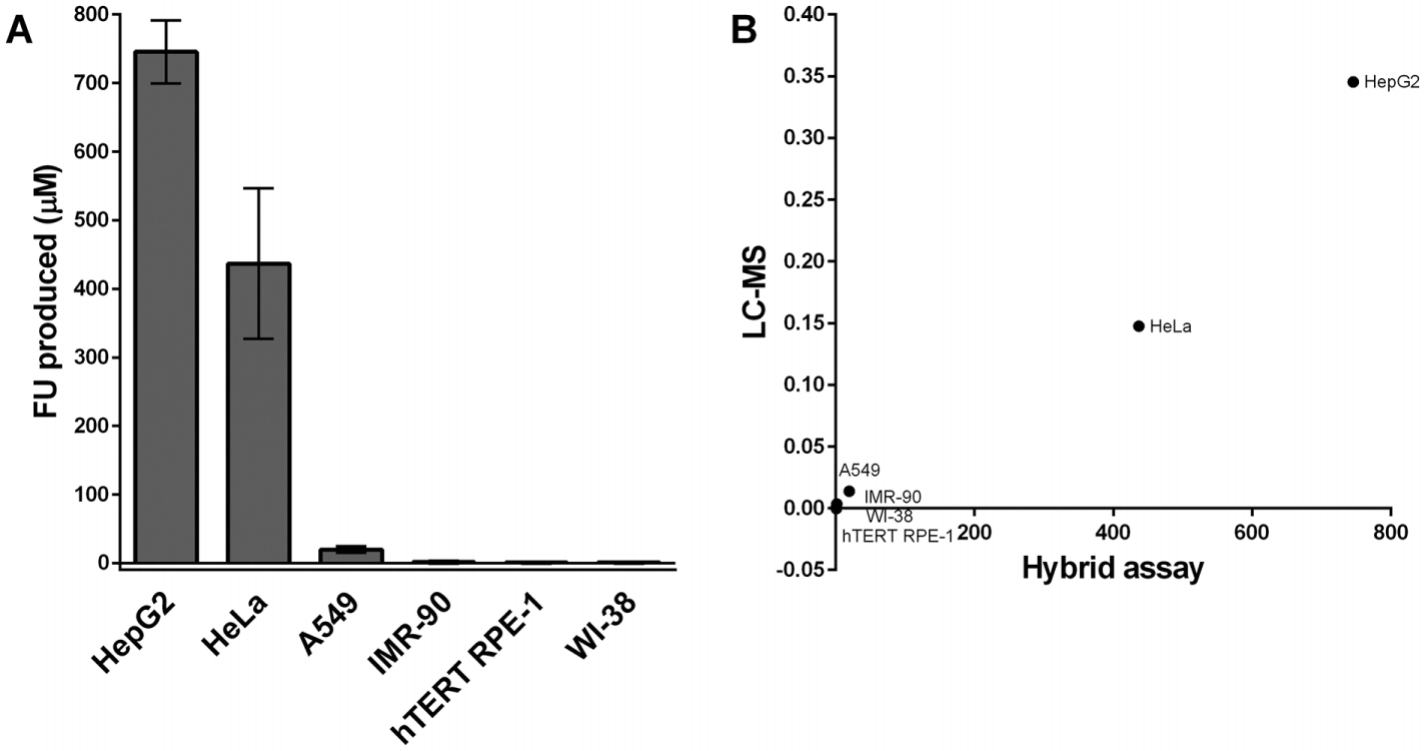
Quantification of CDA activity across a panel of human cell lines. (**A**) The hybrid assay was used to measure the amount of FU produced by cytoplasmic lysates from the indicated cell lines. The data, presented as the mean FU concentration (µM) ± SD from three independent experiments, show a wide range of enzymatic activities among the tested cell lines. (**B**) The relationship between the data obtained from the hybrid assay and the LC-MS method in the same cell lines. The data for the LC-MS analysis were taken from [30].

Using these optimized conditions, we applied the full hybrid assay to quantify CDA activity in cytoplasmic lysates from a diverse panel of human cell lines. The assay revealed a broad dynamic range of activities (Fig. 4A). To determine the status of each individual cell line, we tested whether their measured activity was significantly greater than zero. As detailed in Table 1, the results show high CDA activity in HeLa and HepG2 cells, and a lower level of activity in A549 cells. Conversely, no significant activity was detected in the primary (IMR-90, WI-38) or hTERT RPE-1 cell lines.

**Table 1.** CDA activity and statistical analysis in a panel of human cell lines

Cell line	FU produced (μM)	*P*-value	Result at α = 0.05	Sample size
HepG2	745.45 ± 46.23	.0006	Significant	*n* = 3
HeLa	436.81 ± 109.83	.01	Significant	*n* = 3
A549	19.63 ± 4.68	.0004	Significant	*n* = 3
IMR-90	1.51 ± 2.08	.13	Not significant	*n* = 3
hTERT RPE-1	0.84 ± 1.42	.25	Not significant	*n* = 3
WI-38	0.71 ± 1.17	.25	Not significant	*n* = 3

The table shows the concentration of FU produced from 2 mM FC in 1 h by lysates from different cell lines, presented as mean ± SD. It also summarizes the results of one-sided statistical tests used to determine if the measured CDA activity is significantly greater than zero.

To further validate our hybrid assay, we compared the CDA activities measured in a panel of six cell lines against previously published results obtained using a standard LC-MS method [30]. As shown in Fig. 4B, the activities measured by the hybrid assay showed an exceptionally strong positive correlation with those measured by LC-MS across several orders of magnitude (Pearson coefficient = 0.9768, *P* < .001). This result demonstrates that our cell-based assay provides data that are highly consistent with a gold-standard quantitative method, confirming its suitability for the reliable measurement of CDA activity.

**Figure 5.**
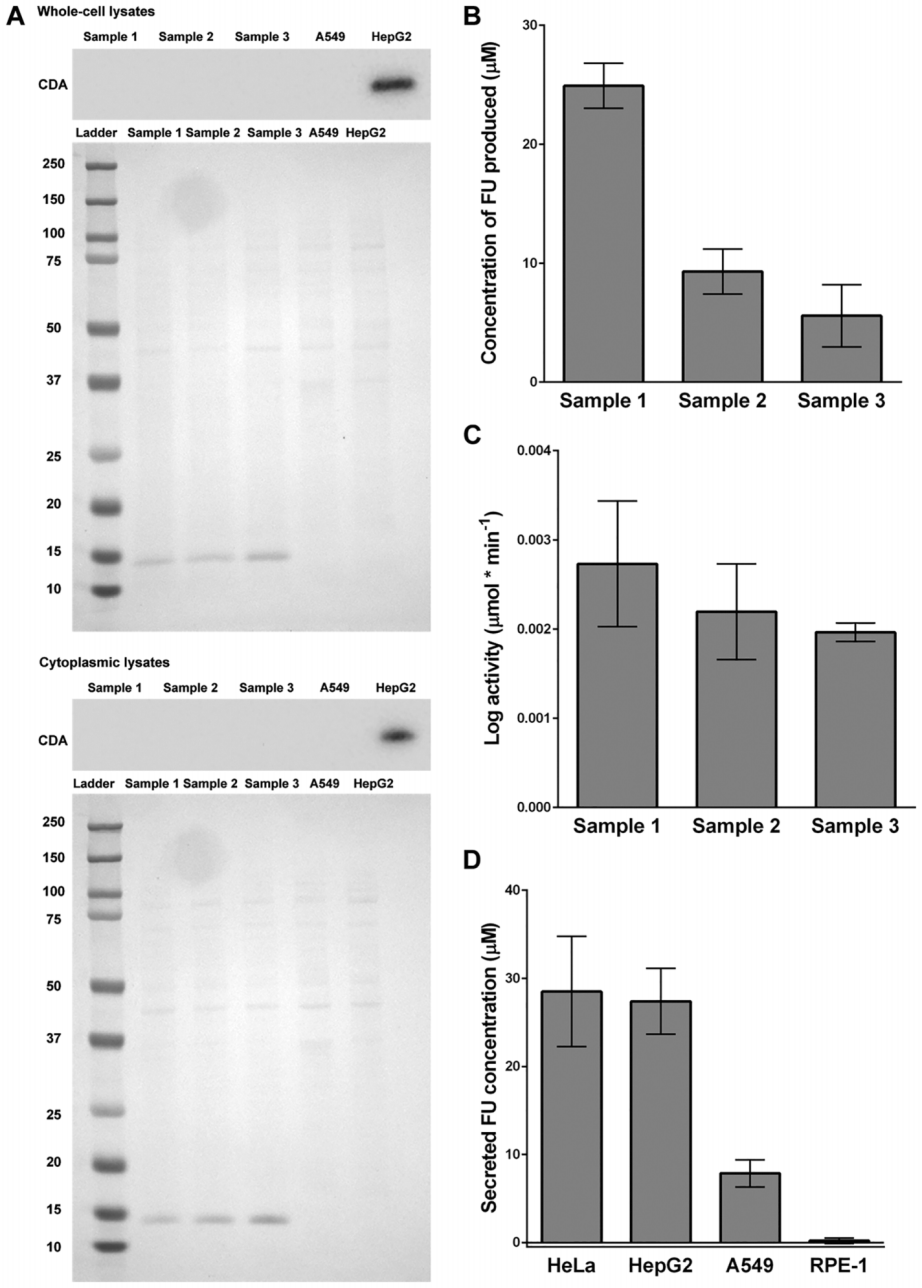
Quantification of CDA amount and activity in PDX model VFN AML2 and detection of secreted FU from CDA-positive live cells. (**A**) Analysis of CDA protein content in three samples derived from VFN AML2 PDX model using western blot. HepG2 cells (high cytidine deaminase levels) serve as a positive control, while A549 cells were included as a low-activity reference. Western blots were performed both on the whole-cell lysates (top) and cytoplasmic lysates (bottom). A representative Ponceau S staining used as a loading control is shown below the corresponding western blots (all individual biological replicates and uncropped blots are available in the Supplementary Data—Originals of Western blots file). (**B**) Analysis of CDA activity in cytoplasmic lysates prepared from tumours of VFN AML2 PDX model using the developed hybrid assay. The graph depicts the concentration of FU produced from FC. The data are shown as the mean ± SD, *n* = 3. (**C**) Analysis of CDA activity in cytoplasmic lysates prepared from tumours of VFN AML2 PDX model using LC-MS assay. The data are shown as the mean ± SD, *n* = 3. (**D**) Conditioned medium from CDA-positive (HeLa, HepG2) and low-activity (A549) cell lines, previously incubated with FC, was transferred to 143B reporter cells. The resulting fluorescence signal indicates the presence of secreted FU in the medium from all CDA-expressing cell lines, in contrast to the CDA-negative hTERT RPE-1 line. The data are shown as the mean ± SD, *n* = 3.

To demonstrate the applicability of our assay on more clinically relevant samples, we next analysed CDA activity in cytoplasmic lysates from a patient-derived xenograft (PDX) model of AML. These samples are often more complex than those from immortalized cell lines, providing a more stringent test of an assay’s robustness. Notably, CDA protein remained undetectable by standard western blot analysis in both the PDX samples and the low-activity A549 cell line, even when using whole-cell lysates (prepared according to [36]) to ensure complete protein extraction (Fig. 5A). In stark contrast, our hybrid assay successfully detected and quantified significant functional CDA activity in the same samples of cytoplasmic lysates (Fig. 5B and Table 2). This demonstrates that our assay can quantify enzymatic activity even when the enzyme concentration is below the detection threshold of western blot analysis. This key finding was validated using an LC-MS, which also confirmed the presence of CDA activity in these samples (Fig. 5C). Taken together, this experiment highlights the superior sensitivity of our functional assay over conventional protein detection methods and confirms its applicability for CDA analysis in complex, patient-derived biological materials.

**Table 2.** CDA activity and statistical analysis in PDX model samples

	FU produced (μM)	*P*-value	Result at α = 0.05	Sample size
Sample 1	24.91 ± 1.89	.0014	Significant	*n* = 3
Sample 2	9.30 ± 1.89	.010	Significant	*n* = 3
Sample 3	5.58 ± 2.61	.047	Significant	*n* = 3

The table shows the concentration of FU measured in samples of PDX model VFN AML2, presented as mean ± SD. It also summarizes the results of one-sided, one-sample t-tests used to determine if the measured concentration is significantly greater than zero.

### Comparison with a direct fluorescent assay highlights the impact of lysate matrix interference

To further validate the utility of our hybrid approach, we compared its performance against a direct, fluorescence-based ammonia detection method (ammonia assay kit) using crude cell lysates. The direct method proved highly susceptible to matrix interference. The optimal lysate concentration used in our hybrid assay (200 µg/ml protein) caused complete signal saturation due to the reaction of the *o*-phthalaldehyde probe with endogenous cellular amines.

To perform the measurement within the kit’s calibration range (0–1 mM ammonium chloride), the lysate concentration had to be reduced four-fold (to 50 µg/ml). Under these conditions, the direct fluorescent assay failed to detect statistically significant CDA activity (ammonia production) following 2 mM FC treatment, both in the highly active HeLa cell line (paired t-test; *P* = .5112) and the low-activity A549 line (paired t-test; *P* = .2206, Supplementary Fig. S3). Furthermore, the LOD of the formed product (ammonia) was severely degraded by the lysate matrix, increasing ~5-fold from 6.75 µM (for pure ammonia in buffer) to 35.75 µM (in the lysate matrix). This comparative analysis underscores that while direct fluorescent assays are highly sensitive in pure solutions, their efficacy is severely compromised by the complex matrix of crude biological lysates, emphasizing the advantage of the physical separation step in our hybrid assay.

### CDA-positive cells actively secrete the metabolic product FU into the culture medium

Having established a robust method for measuring CDA activity in cell lysates, we next investigated whether the product FU is secreted by intact cells. To test this, we incubated various cell lines with FC and measured the concentration of FU in the conditioned medium. The results revealed that CDA-expressing cell lines release FU into their environment (Fig. 5D). The exact concentrations and statistical confirmation that this release is significantly greater than zero are provided in Table 3.

**Table 3.** Concentration of secreted FU and statistical analysis

Cell line	Secreted FU concentration (μM)	*P*-value	Result at α = 0.05	Sample size
HeLa	28.50 ± 6.26	.0008	Significant	*n* = 3
HepG2	27.39 ± 3.74	.0002	Significant	*n* = 3
A549	7.86 ± 1.55	<.0001	Significant	*n* = 3
hTERT RPE-1	0.20 ± 0.31	.1430	Not significant	*n* = 3

The table shows the concentration of FU measured in the culture medium after 1-h incubation with different cell lines, presented as mean ± SD. It also summarizes the results of one-sided, one-sample t-tests used to determine if the measured concentration is significantly greater than zero.

We specifically designed our secretion assay with a brief 1-h incubation to preclude the kinetic window of 5-FU-induced apoptosis and subsequent secondary necrosis. Nevertheless, to confirm that the detected FU originates from active secretion rather than passive leakage from dead or dying cells, we evaluated cell viability using an Annexin V/PI assay. All four cell lines were either treated with 100 μM FC or left untreated (control samples) for 1 h. Quantitative image analysis of over 4500 cells per condition revealed no statistically significant increase in the fraction of apoptotic (Annexin V-positive), necrotic (PI-positive), or late apoptotic cells compared to untreated controls (Supplementary Table S1). Further, while conditioned medium harvested from an overnight culture of HeLa cells did show statistically significant CDA activity, this signal accounted for only ∼7% of the total signal generated by intact cells in the secretion assay (*P* = .009, independent t-test). These results confirm that the vast majority of the detected FU is produced intracellularly and subsequently exported from viable cells. Furthermore, we correlated the FU production capacity of multiple cell lines with their cell death stages (Table 4). If the passive leakage hypothesis were correct, the high-producing cell lines should exhibit extensive cell death. However, our data do not support this assumption. The highly efficient converter HepG2 (producing 745.45 μM FU) showed near-zero necrosis (0.06%) and late apoptosis (0.15%). Paradoxically, the non-producing RPE-1 cells (0.84 μM FU) displayed numerically higher necrosis (1.24%). We also performed a Pearson correlation analysis between the amount of FU produced and the percentages of all three stages of cell death. The analysis revealed a non-significant inverse relationship for necrosis (r = −0.925, *P* = .075) and a moderate inverse non-significant relationship for late apoptosis (r = −0.590, *P* = .410). The complete absence of any positive correlation between FU production and membrane-permeable stages of cell death definitively refutes the assumption that higher FU accumulation drives secondary necrosis.

**Table 4.** Correlation between 5-FU production and cell death stages

Cell line	FU produced (μM)	Annexin V (%)	Annexin V/PI (%)	PI (%)
HepG2	745.45 ± 46.23	0.43 ± 0.14	0.15 ± 0.23	0.06 ± 0.09
HeLa	436.81 ± 109.83	1.23 ± 0.38	0.69 ± 0.48	0.29 ± 0.01
A549	19.63 ± 4.68	0.13 ± 0.19	0.77 ± 0.68	0.77 ± 1.17
hTERT RPE-1	0.84 ± 1.42	0.16 ± 0.30	0.41 ± 0.25	1.24 ± 0.55

To put this active secretion into a physiological context, we compared it to the total cellular FU production capacity in HeLa cells. Based on our direct measurement of 132.3 ± 0.005 pg of protein per cell, the 200 µg of protein used in our lysate assays corresponds to ~1 512 000 cells. The concentration of 436.81 µM FU produced in the lysate assay (Table 1) translates to a total production capacity of ~289 nmol of FU per million cells per hour. For the secretion assay, the measured concentration of 28.50 µM in 2 ml of medium (Table 3) corresponds to a total secreted amount of 57 nmol of FU per million cells per hour. Comparing these two values, we conclude that live HeLa cells actively secrete ~19.7% of the total FU they are capable of producing. However, it is important to note that this comparison serves as a rough estimate, as it relies on the assumption that the enzymatic activity of CDA in the optimized conditions of the cell lysate closely reflects its activity within the complex intracellular environment of live cells. Nevertheless, this observation provides direct evidence for a potential mechanism for a metabolic bystander effect in a heterogeneous cell population. On a single-cell level, this equates to a total production capacity of ~289 fmol per cell per hour, from which 57 fmol are actively secreted.

## Discussion

In this study, we aimed to address the persistent challenges in quantifying CDA activity. We successfully developed and established a novel, robust hybrid assay that functions reliably in complex biological samples. Our key findings are two-fold: first, the development of a reproducible and cost-effective method that occupies a unique niche among existing assays; and second, the important biological observation that CDA-expressing cells actively secrete the metabolic product, FU, into their extracellular environment.

The primary advantage of our method is its robustness in complex biological matrices. This design principle addresses a key limitation of the simplest and most cost-effective techniques, the spectrophotometric assays. While straightforward, direct spectrophotometric methods are highly susceptible to interference from other components in crude lysates, such as nucleotides and proteins, which can absorb at similar wavelengths [45, 46]. Similar obstacles accompany indirect methods based on the release of ammonia during cytidine deamination [47, 48]. Interference from ammonia originating from numerous other metabolic pathways is a significant problem, as it can lead to a high background that masks the specific signal from CDA. It is therefore essential to use carefully designed controls for such assays. Our direct comparative testing with a commercial fluorescence-based ammonia assay (utilizing the *o*-phthalaldehyde method) confirmed these severe limitations. While highly sensitive in pure buffer (LOD ∼6.75 µM), its application to crude cell lysates required the avoidance of optimal Tris-based buffers and suffered from massive background signal from endogenous cellular components. The assay consequently failed to detect significant CDA activity even in highly active HeLa lysates, with the LOD degrading ~5-fold due to the matrix effect. By physically separating the enzymatic reaction from the highly specific detection step, our hybrid method effectively bypasses these matrix interferences, allowing for robust quantification without sample purification. Our hybrid method circumvents these problems entirely. Its specificity is conferred by the highly selective response of the reporter cells to the FU product alone, and a cornerstone of this reliability is the rigorous selection of the 143B reporter line. After excluding primary cell lines due to their limited lifespan, we demonstrated that the 143B line is not only functionally CDA-negative but also significantly more sensitive than the immortalized hTERT-RPE-1 line, ensuring a high signal-to-noise ratio and eliminating the need for extensive sample purification.

A more nuanced advantage in terms of robustness is evident even when comparing our assay to the gold-standard method, LC-MS. Although LC-MS is not susceptible to the spectral interferences that affect spectrophotometry, it is known to suffer from the matrix effect. Components of the crude lysate, such as non-volatile salts like Tris, can interfere with the ionization process and compromise accurate quantification, often necessitating extensive sample purification. Our hybrid assay avoids this specific type of interference, as the reporter cells are not affected by such components at typical working concentrations.

Naturally, any cell-based system is sensitive to cytotoxic agents, including detergents or high concentrations of salts. However, this potential issue is effectively mitigated by the stepwise dilution process inherent to our protocol. First, the initial crude lysate is normalized to a standard working concentration (200 µg/ml), which often involves a significant dilution of the original concentrated sample. Subsequently, the heat-inactivated supernatant is further diluted 1:1 with culture medium before being applied to the reporter cells. This cumulative dilution effectively reduces the concentration of any potentially cytotoxic components from the lysis buffer to well-tolerated levels. Thus, the assay’s design successfully circumvents both the spectral interferences of spectrophotometry and the ionization interferences of LC-MS, positioning it as a highly robust tool for use with crude biological samples.

A balanced assessment of our assay’s capabilities is crucial. The sensitivity, with a detection limit of ~2.7 µM FU, is comparable to that of standard absorbance-based spectrophotometric methods. This is substantially less sensitive than high-end methods like LC-MS or radiometric assays, which can achieve nanomolar or even picomolar detection limits [30, 49, 50]. This positions our assay as an ideal tool for reliably quantifying moderate to high levels of CDA activity, rather than for applications requiring the detection of trace enzymatic activity.

From a practical standpoint, our assay offers a compelling balance of cost, complexity, and throughput. The estimated cost per sample is low (<1 EUR), comparable to spectrophotometry, and dramatically lower than LC-MS, which can range from 5 to over 10 EUR per sample. It also avoids the need for the highly specialized equipment and personnel required for mass spectrometry. Moreover, its workflow is readily adaptable to a multi-well plate format, making it highly suitable for high-throughput screening (HTS). This arrangement could further decrease the cost per sample, and the procedure could be significantly shortened if the fluorescence is measured directly with plate readers instead of microscopes. Such a format is particularly valuable for preclinical pharmacological applications, such as screening large small-molecule libraries for novel CDA inhibitors or profiling functional CDA activity across extensive panels of patient-derived xenografts or cancer cell lines. It is important to emphasize that unlike quantitative immunoassays (such as ELISA), which merely measure protein abundance, our assay provides a direct readout of functional catalytic activity. As demonstrated by our PDX model data, significant enzymatic activity can be present even when protein levels fall below the detection limits of antibody-based methods, underscoring the critical need for functional activity assays in pharmacological research.

Perhaps one of the most significant findings of this study is the clear demonstration that CDA-expressing cells actively secrete the product FU into the culture medium. This observation provides direct evidence for a potential metabolic bystander effect. FU produced within one cell is not confined but can act on neighbouring cells within a heterogeneous tumour microenvironment. This could have profound consequences for therapies involving prodrugs like capecitabine that are intracellularly converted to dFU, a process in which CDA plays a vital role [13, 14]. This bystander effect could potentially enhance the therapeutic window by killing adjacent tumour cells that did not metabolize the prodrug, but it could also increase local toxicity to healthy tissue, a known issue in cancer therapy.

In addition to our primary findings, this study provides several practical benchmarks for researchers. Our lysis protocol yields a consistent cytoplasmic protein amount of ~132.3 pg per HeLa cell. From this, we calculated a total FU production capacity of ~289 fmol per cell per hour. We then determined that live cells actively secrete ~19.7% of this capacity. These quantitative values can serve as useful reference points for future studies in drug metabolism and cell biology.

In conclusion, we have developed a hybrid assay that successfully fills a gap in the portfolio of available methods for CDA quantification. While not as sensitive as LC-MS, it combines the low cost and simplicity of spectrophotometry with the crucial ability to function robustly in crude biological lysates. This, coupled with its suitability for HTS, makes it a powerful new tool for screening CDA activity in a wide range of research applications, from fundamental enzymology to pharmacology.

## Supplementary Material

gkag588_Supplemental_Files

## Data Availability

All data are included in this article and in the Supplementary data files. Additional data supporting the findings are available from the corresponding authors upon reasonable request. The SIMCIM tools software can be downloaded from https://doi.org/10.5281/zenodo.20409591.
