# Supplementary material for: A hybrid RNA-based reporter assay for robust quantification of cytidine deaminase activity
Source: Nucleic Acids Res. 2026 Jun 11;54(11):gkag588. doi: 10.1093/nar/gkag588 (PMC13254536; doi:10.1093/nar/gkag588)
Supplement: gkag588_Supplemental_Files [file gkag588_supplemental_files.zip › Supplementary Data.pdf]

A hybrid RNA-based reporter assay for robust quantification of cytidine deaminase activity

Anna Ligasová[1*], Martina Horejšová[1, 2], David Friedecký[2], Eva Pokorná[3], Pavel Klener[3], Karel Koberna[1*]

Supplementary Figures and Tables

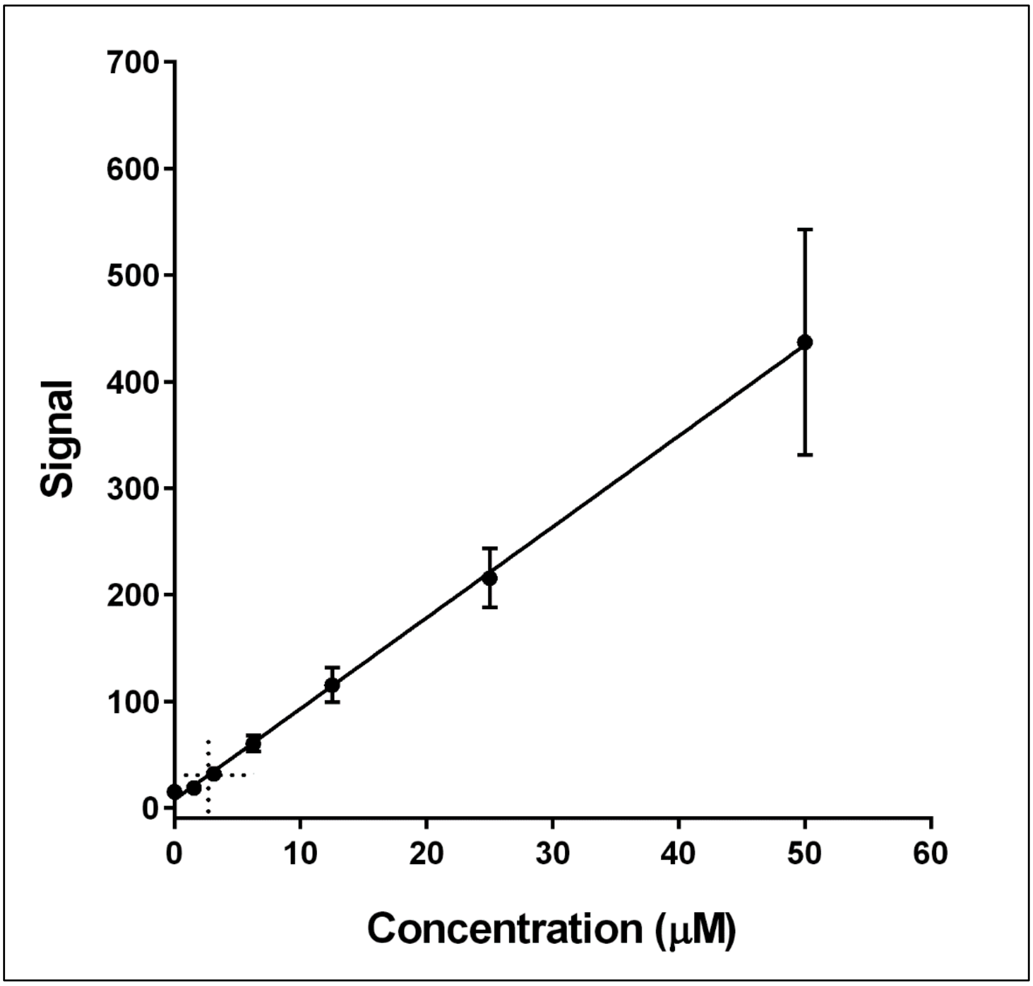

Supplementary Figure 1. Calibration curve used for LOD calculation of the formed product (FU). For the calculation of LOD, data from experiment in which 143B cells were incubated with various concentrations of FU (0-50 μM) for 1 hour. The data are shown as the mean ± SEM, n = 4.

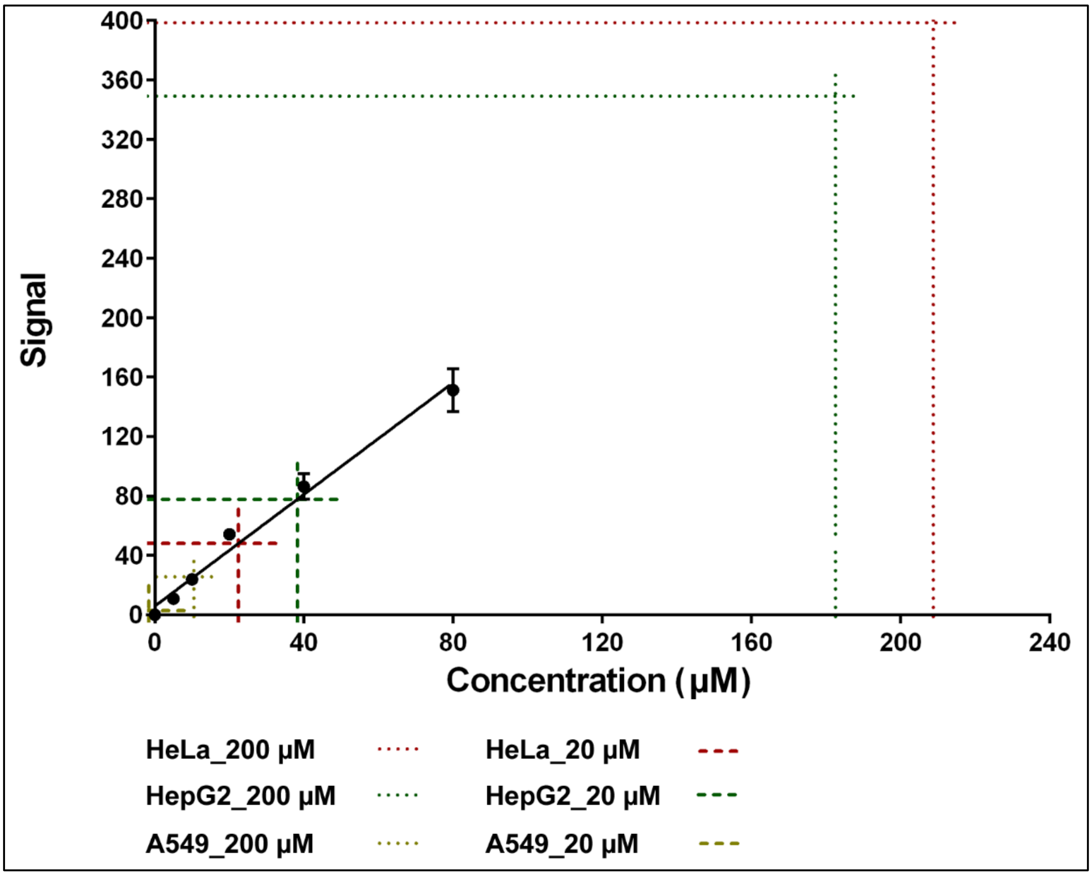

Supplementary Figure 2. Validation of the dual-analysis strategy for samples with diverse CDA activities. Quantitative comparison of FU production at two different lysate protein concentrations (200 μg/mL and 20 μg/mL). For the low-activity A549 line, the 200 μg/mL concentration is necessary to achieve a robust signal. For the high-activity HeLa line, 200 μg/mL results in a signal exceeding the validated calibration range, whereas a 10-fold dilution (20 μg/mL) brings the enzymatic product back into the linear window (up to 80 μM) for accurate quantification. Data are presented as mean ± SD, n = 6.

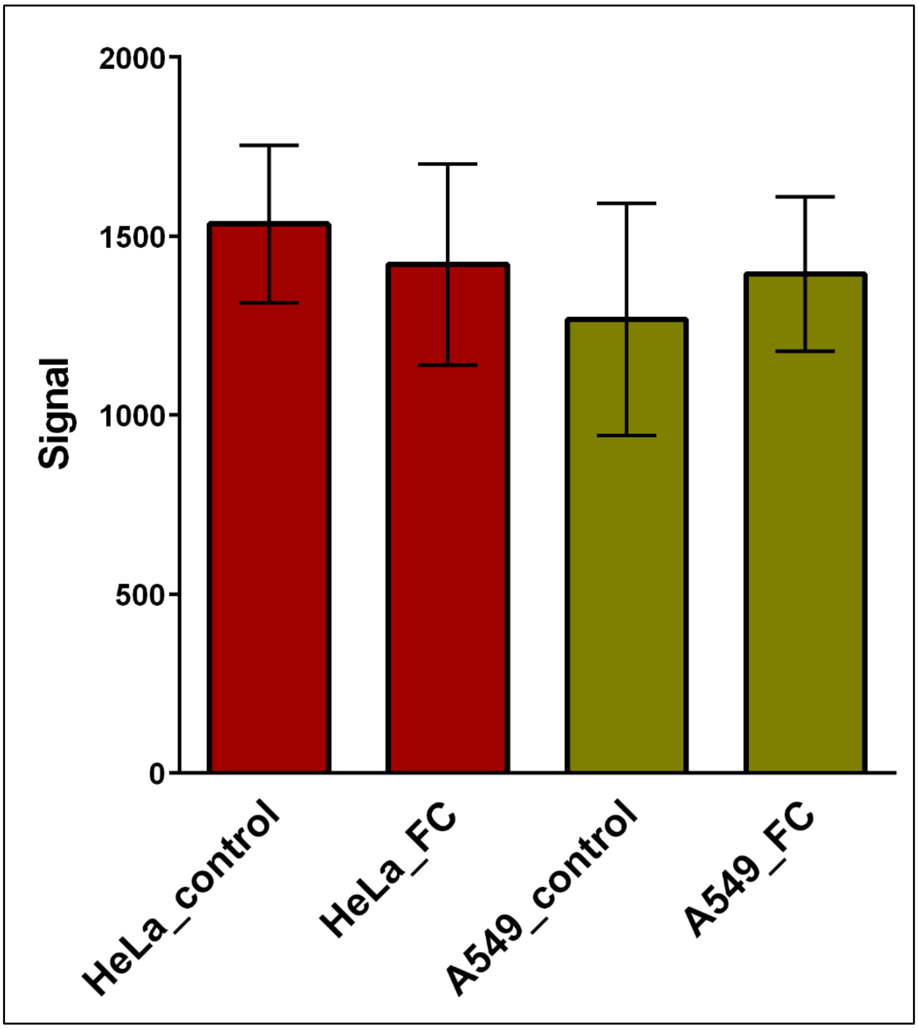

Supplementary Figure 3. Analysis of CDA activity using ammonia assay kit (Merck, MAK310) in cell lysates of HeLa and A549 cells. Data are presented as mean ± SD, n = 3.

Supplementary Table 1 Statistical analysis of the apoptotic (Annexin V-positive), necrotic (PI-positive), or late apoptotic (Annexin V/PI-positive) cells compared to untreated controls.

	**Annexin V**	**Annexin V/PI**	**PI**	**Sample size**
**Cell line**	**p-value**			
HeLa	0.941	0.207	0.612	n = 3
HepG2	0.690	0.126	0.831	n = 3
A549	0.796	0.156	0.979	n = 4
hTERT RPE-1	0.351	0.261	0.358	n = 3
