# Supplementary material for: A hybrid RNA-based reporter assay for robust quantification of cytidine deaminase activity
Source: Nucleic Acids Res. 2026 Jun 11;54(11):gkag588. doi: 10.1093/nar/gkag588 (PMC13254536; doi:10.1093/nar/gkag588)

# A hybrid RNA-based reporter assay for robust quantification of cytidine deaminase activity

Anna Ligasová[1*], Martina Horejšová[1, 2], David Friedecký[2], Eva Pokorná[3], Pavel Klener[3], Karel Koberna[1*]

Originals of Western blots

**Originals of Western blots**

**Figure 2B**

Ponceau S_1

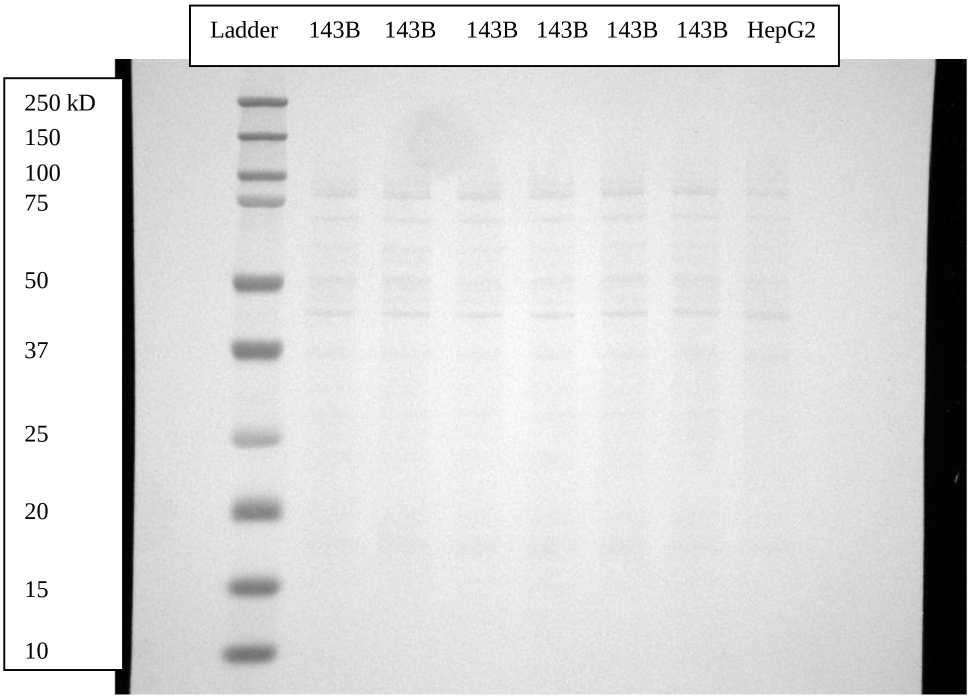


CDA_1

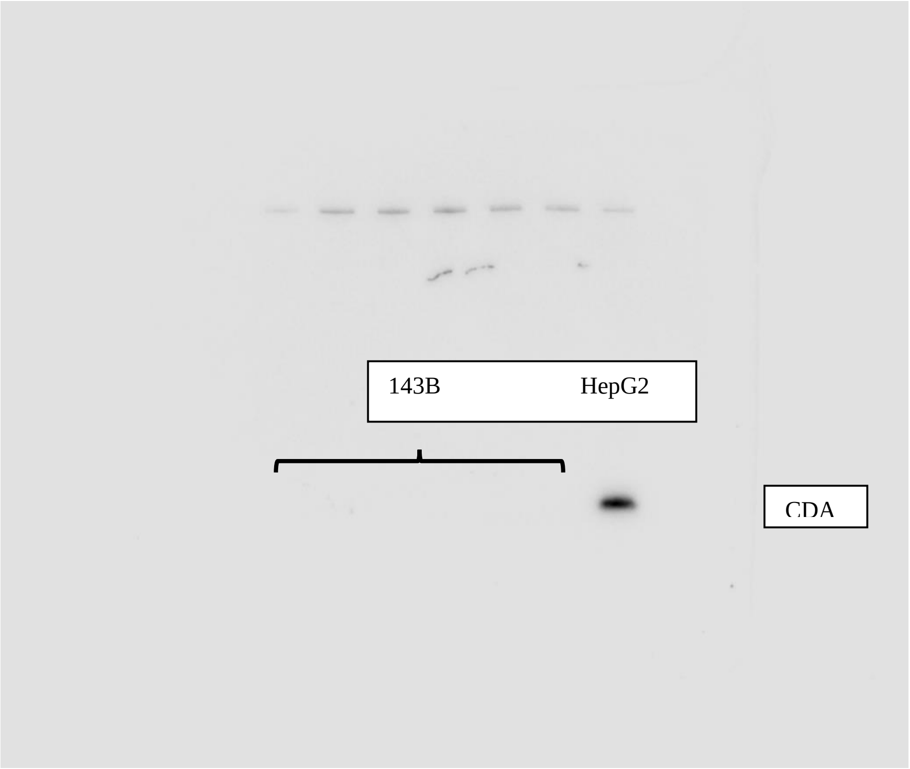

Ponceau S_2

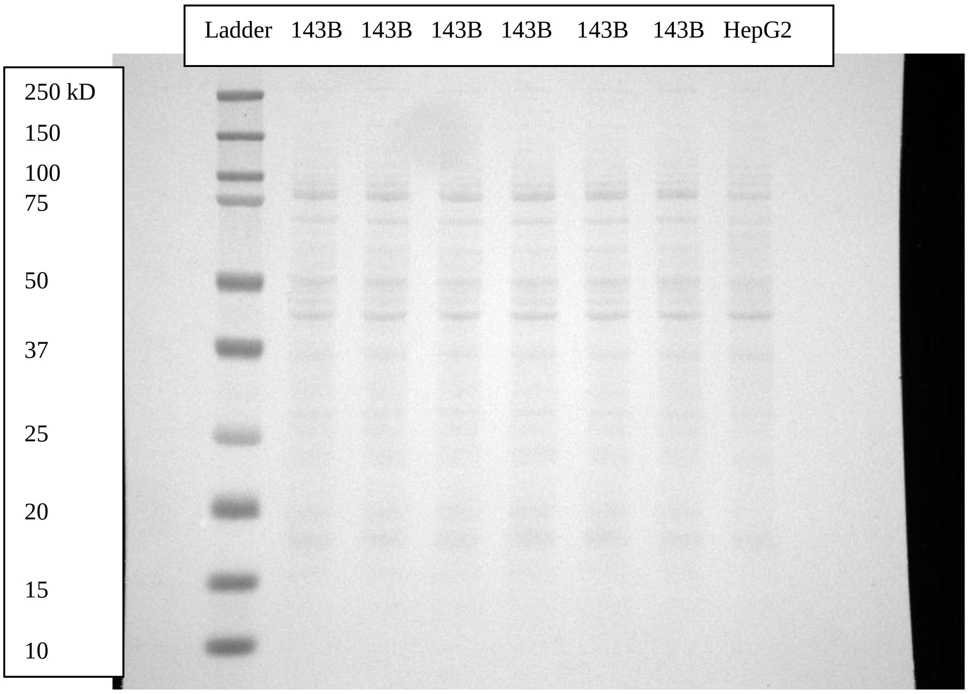


CDA_2

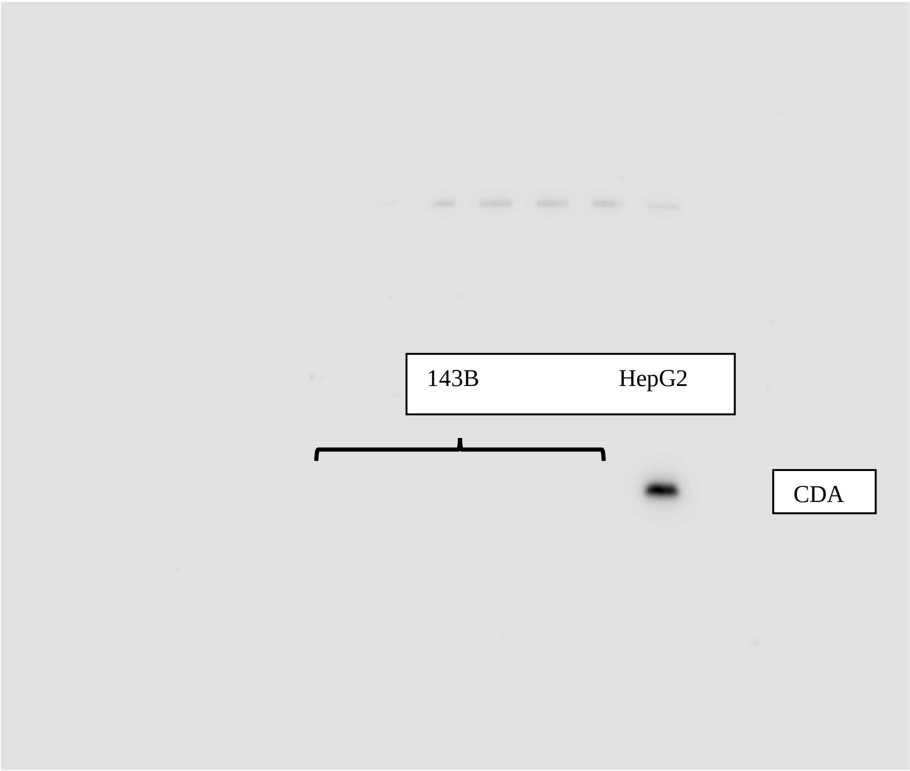

Ponceau S_3

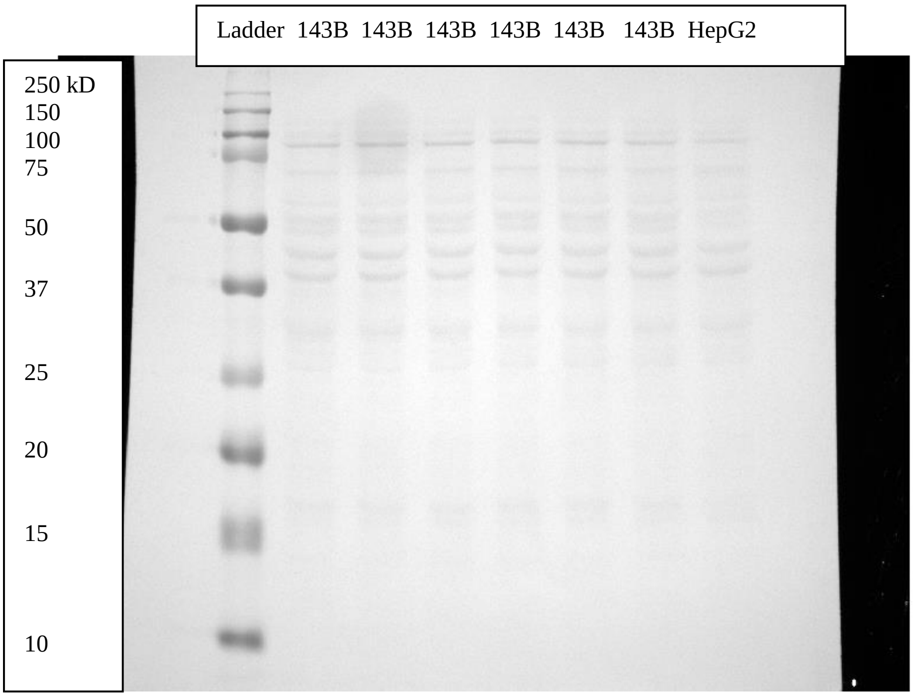


CDA_3

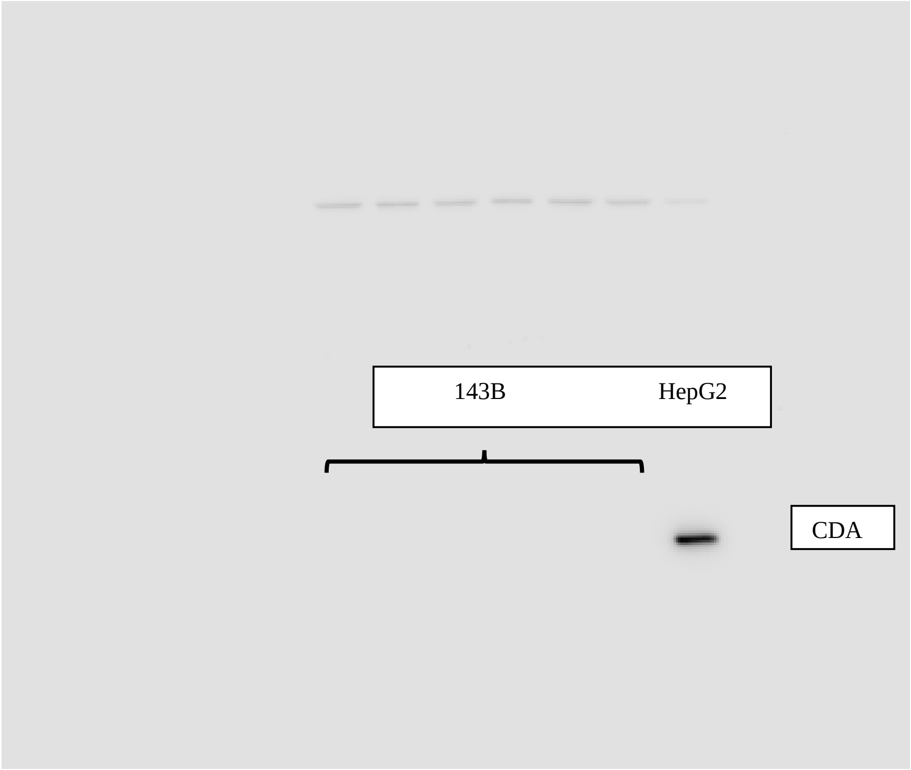

**Figure 5A**

**Whole-cell lysates**

1 =  sample 1; 2 = sample 2; 3 = sample 3

Ponceau S_1

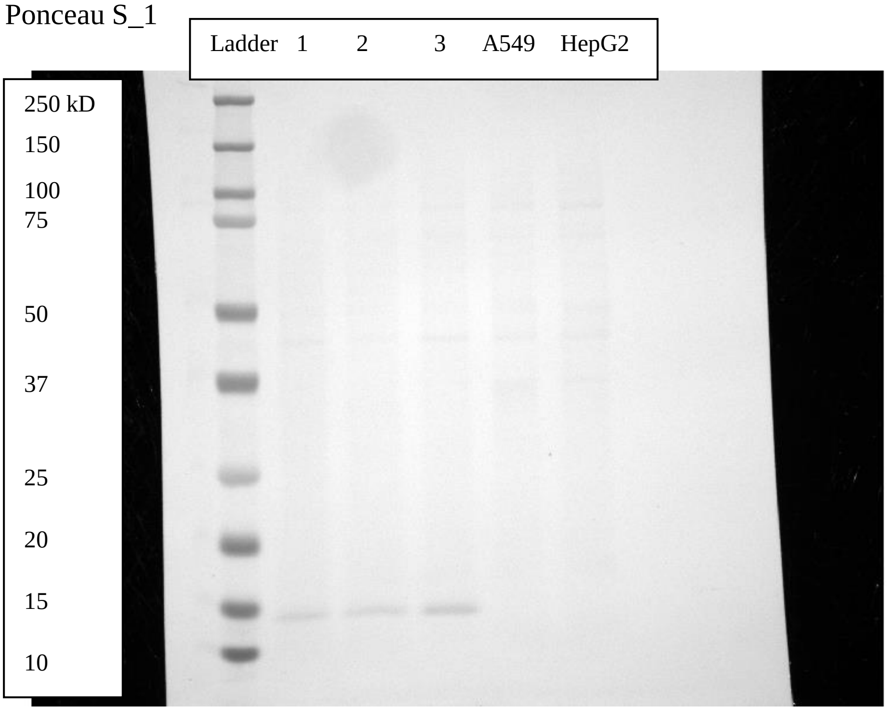


CDA_1

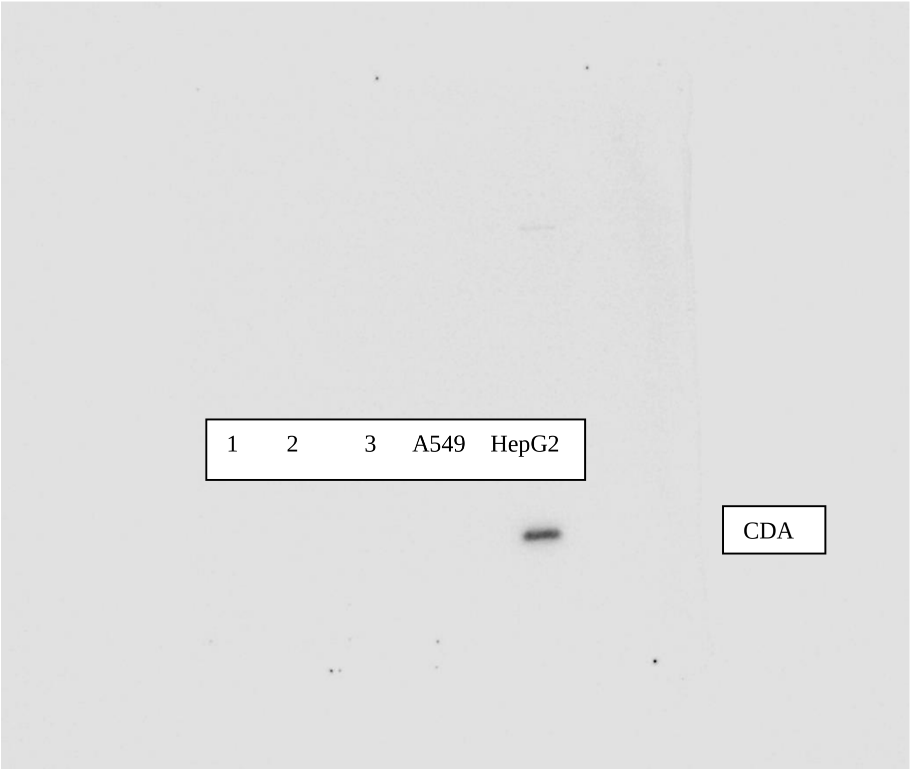

Ponceau S_2

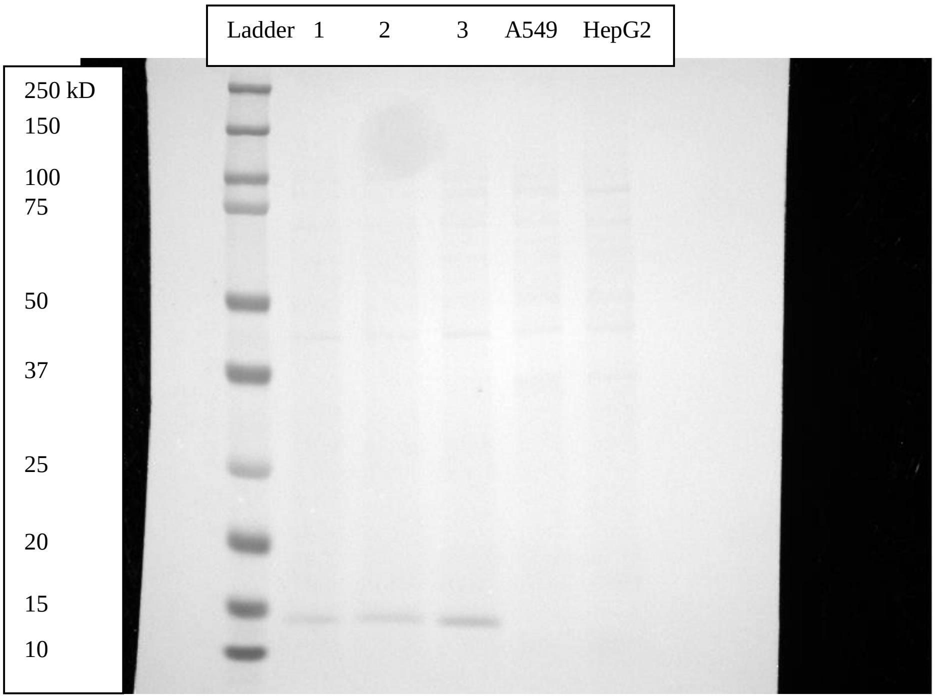


CDA_2

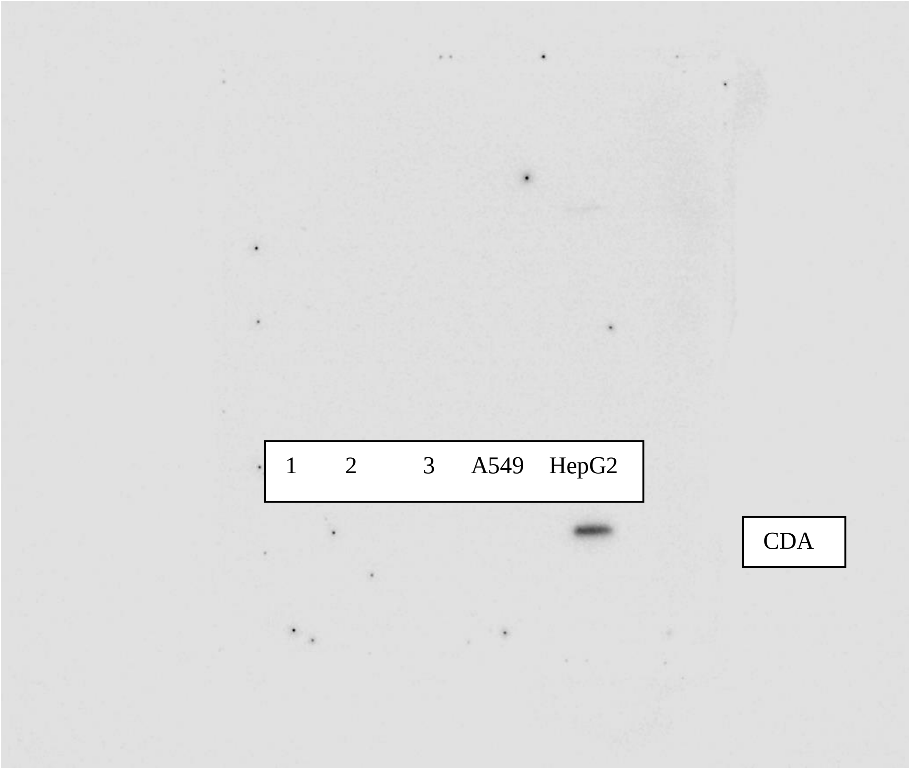

Ponceau S_3

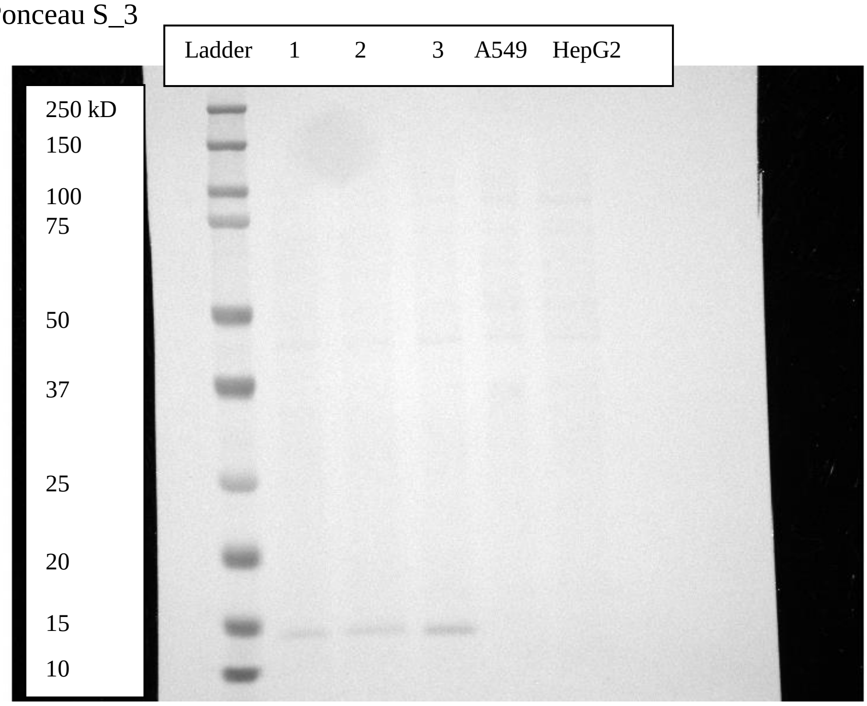


CDA_3

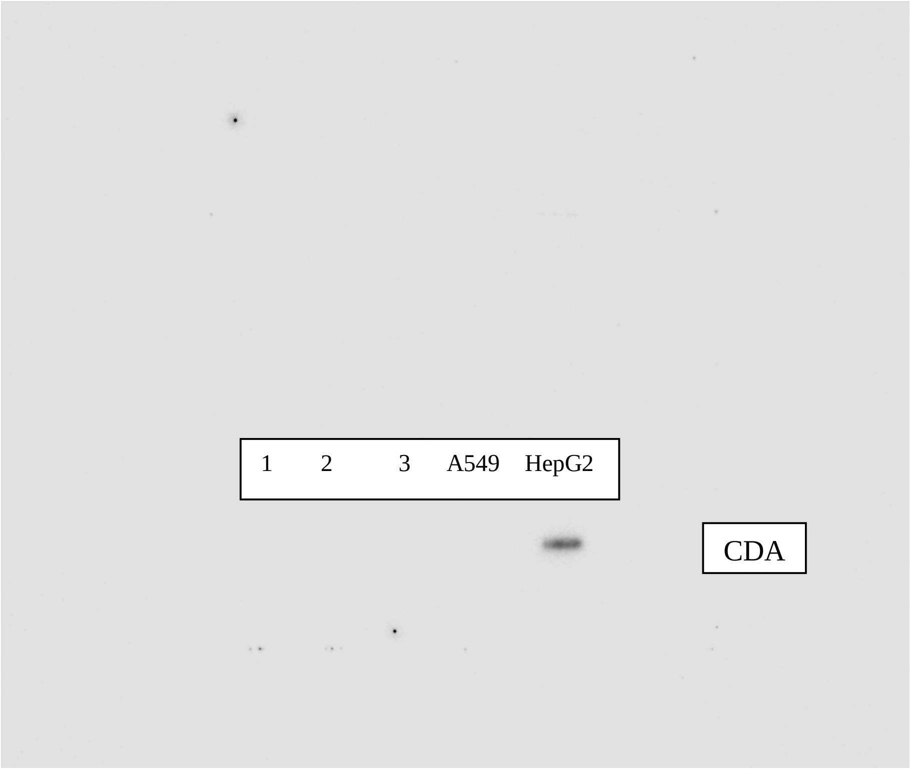

**Cytoplasmatic lysates**

1 = sample 1; 2 = sample 2; 3 = sample 3

Ponceau S_1

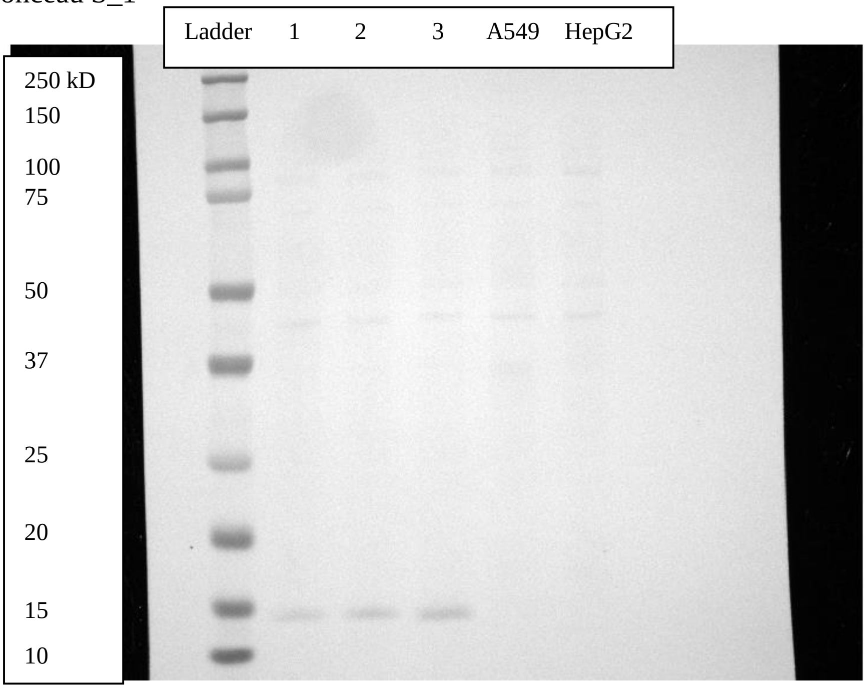


CDA_1

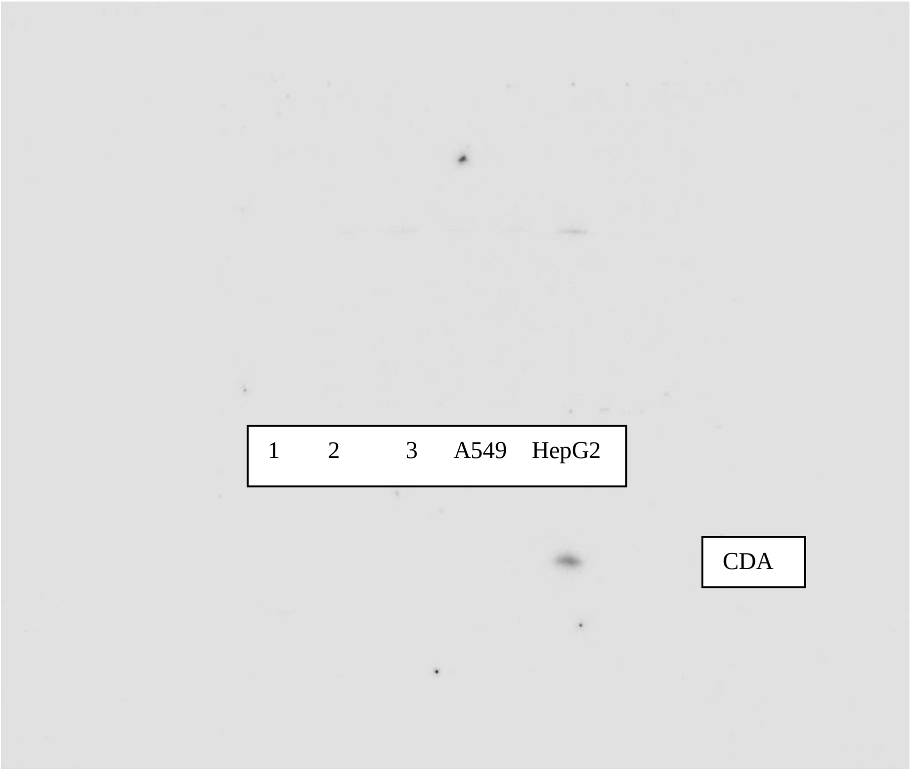

Ponceau S_2

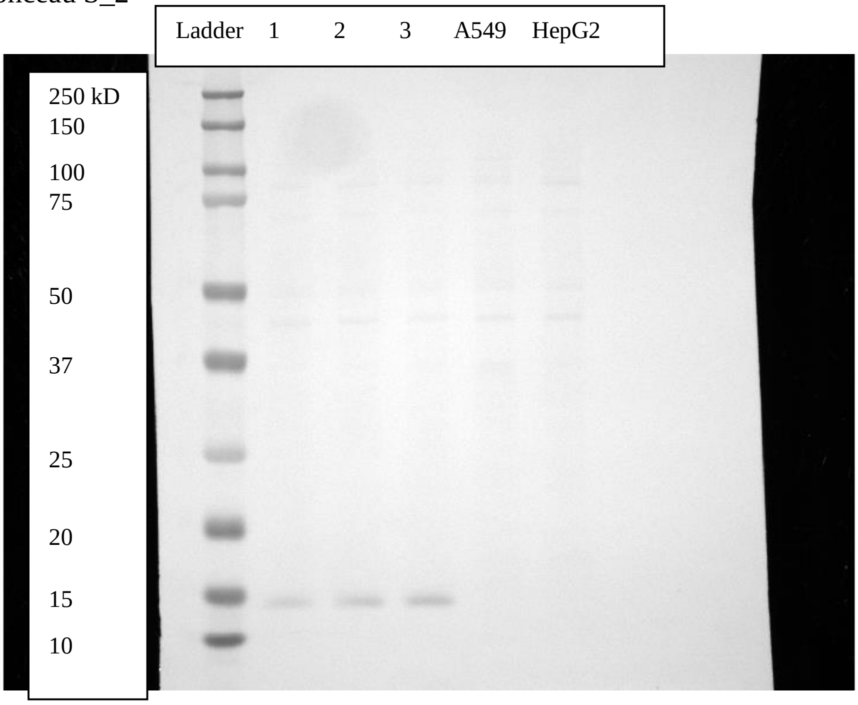


CDA_2

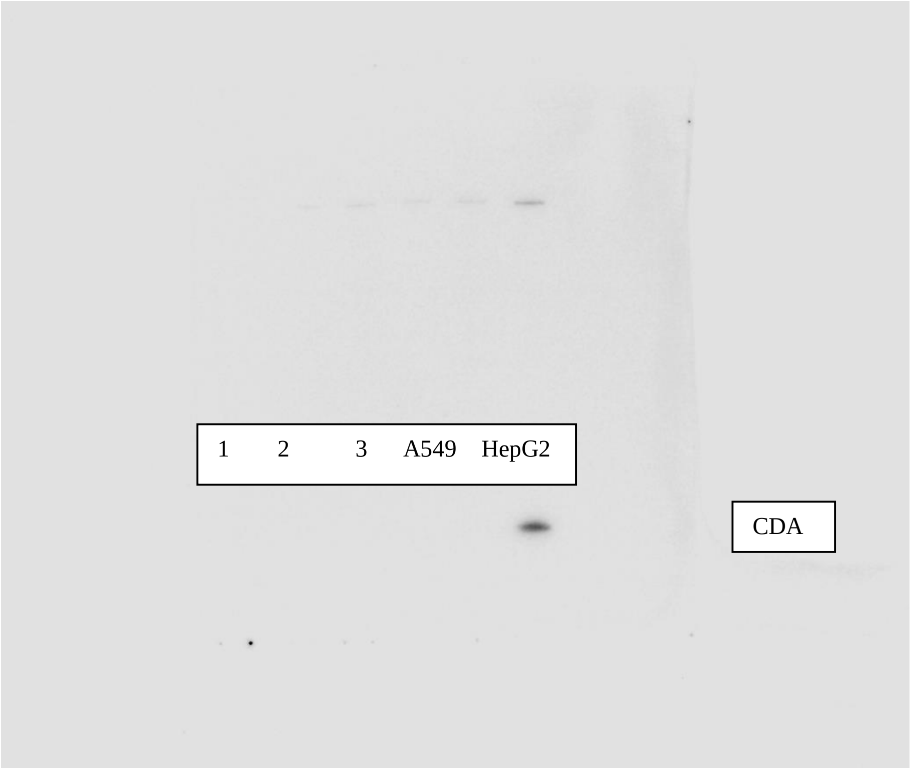

Ponceau S_3

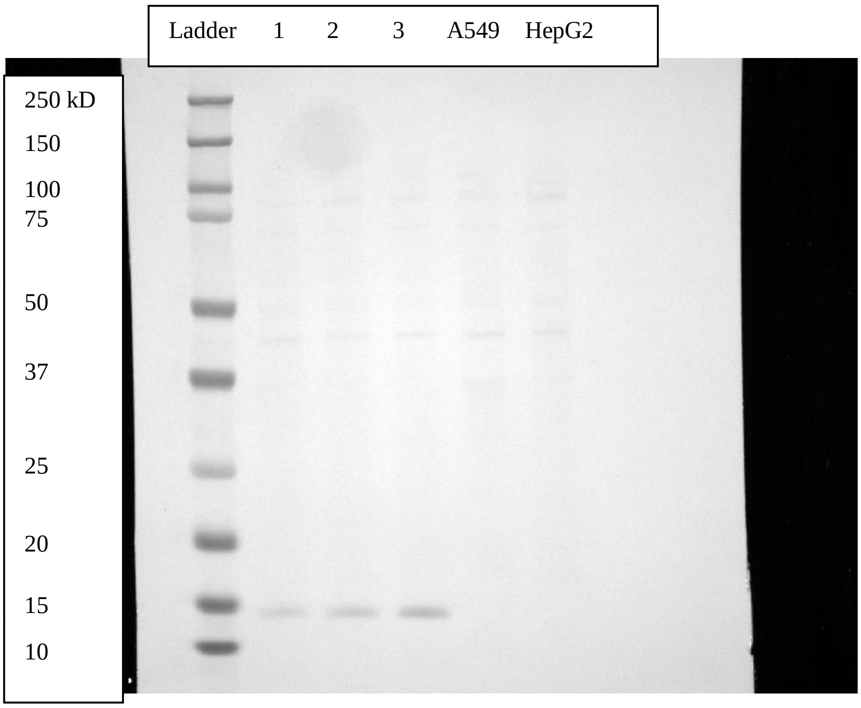


CDA_3

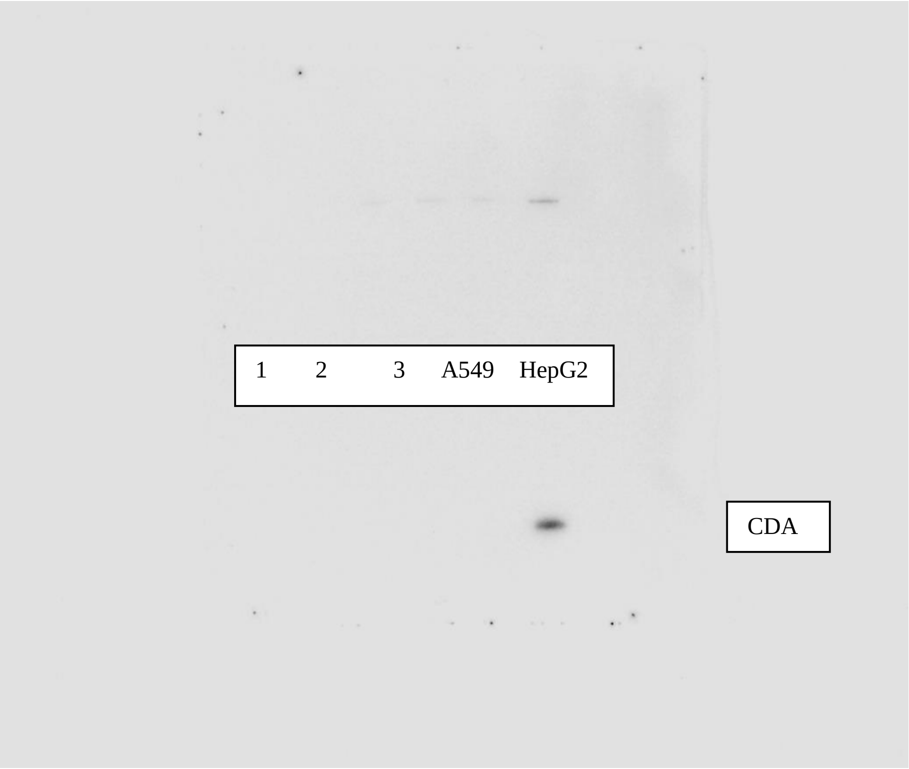

Supplement: gkag588_Supplemental_Files [file gkag588_supplemental_files.zip › Supplementary Data_Originals of Western blots.pdf]
